# Supplementary material for: Netrin-1 Confines Rhombic Lip-Derived Neurons to the CNS
Source: Cell Rep. Author manuscript; Available in PMC 2018 Mar 30. (PMC5877811; doi:10.1016/j.celrep.2018.01.068)
Supplement: 1 [file NIHMS943889-supplement-1.pdf]

Cell Reports, Volume *22*

Supplemental Information

Netrin-1 Confines Rhombic

Lip-Derived Neurons to the CNS

Andrea R. Yung, Noah R. Druckenbrod, Jean-François Cloutier, Zhuhao Wu, Marc Tessier-Lavigne, and Lisa V. Goodrich

Supplemental Information

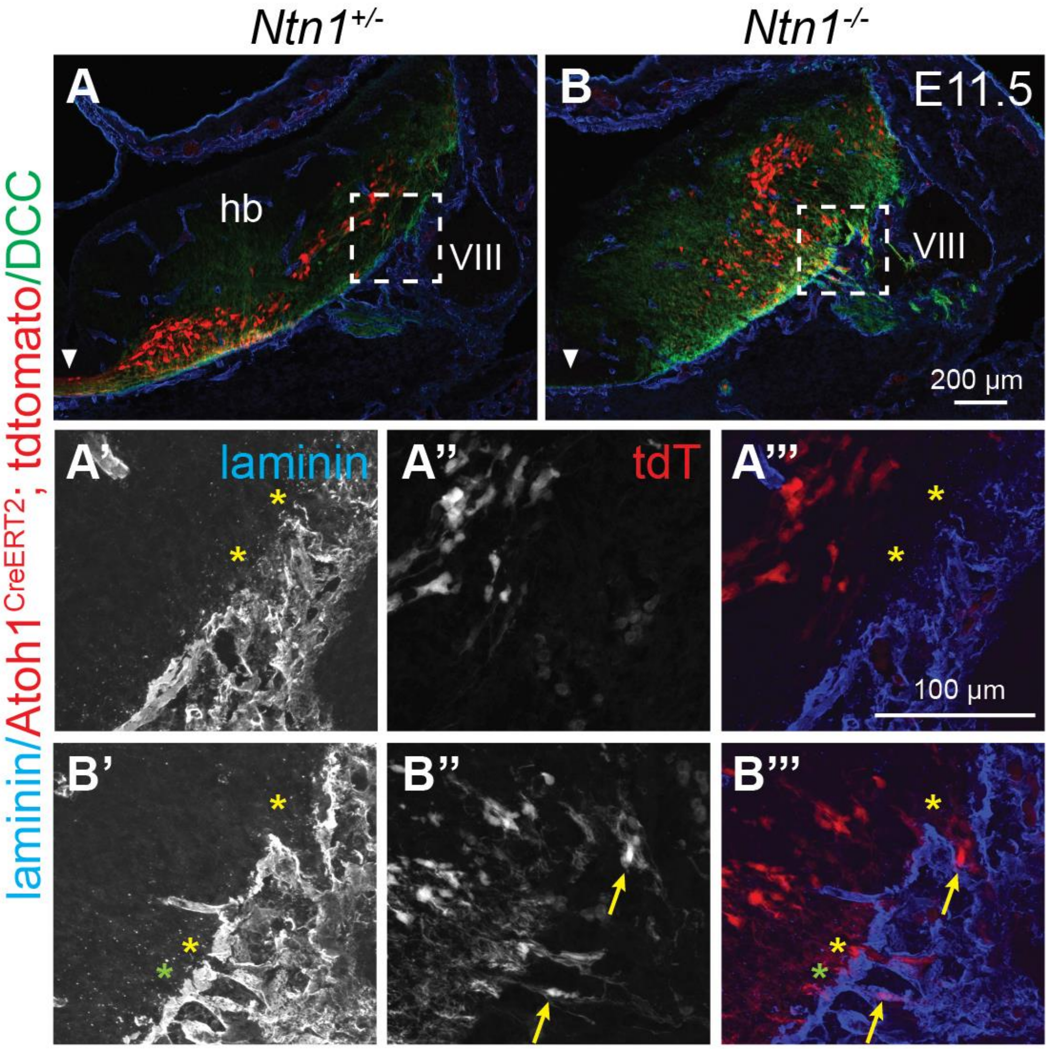

Figure S1: Earlier born rhombic lip neurons exit the CNS at cranial nerve roots in the absence of *Ntn1*. Related to Figure 2. (A-B''') Immunostains of E11.5 transverse head sections from animals that were injected with tamoxifen at E9.5. Low power images of laminin (blue) and tdTomato (red) show that in the absence of Ntn1, commissural neurons fail to form a ventral commissure (white arrowhead) and are located more dorsally (A, B). In control animals (A-A'''), tdTomato+ neurons do not take advantage of weaker areas of BM integrity near nerve entry zones (yellow asterisks, A'-A'''). In contrast, in mutants (B-B'''), a number of processes and cell bodies are observed migrating through the BM (yellow arrows), generating *de novo* breaks in laminin (yellow asterisks). In some cases, neurons traverse the BM before a clear break is observed (green asterisk, B'-B'''). Hb, hindbrain; VIII, vestibulocochlear nerve.

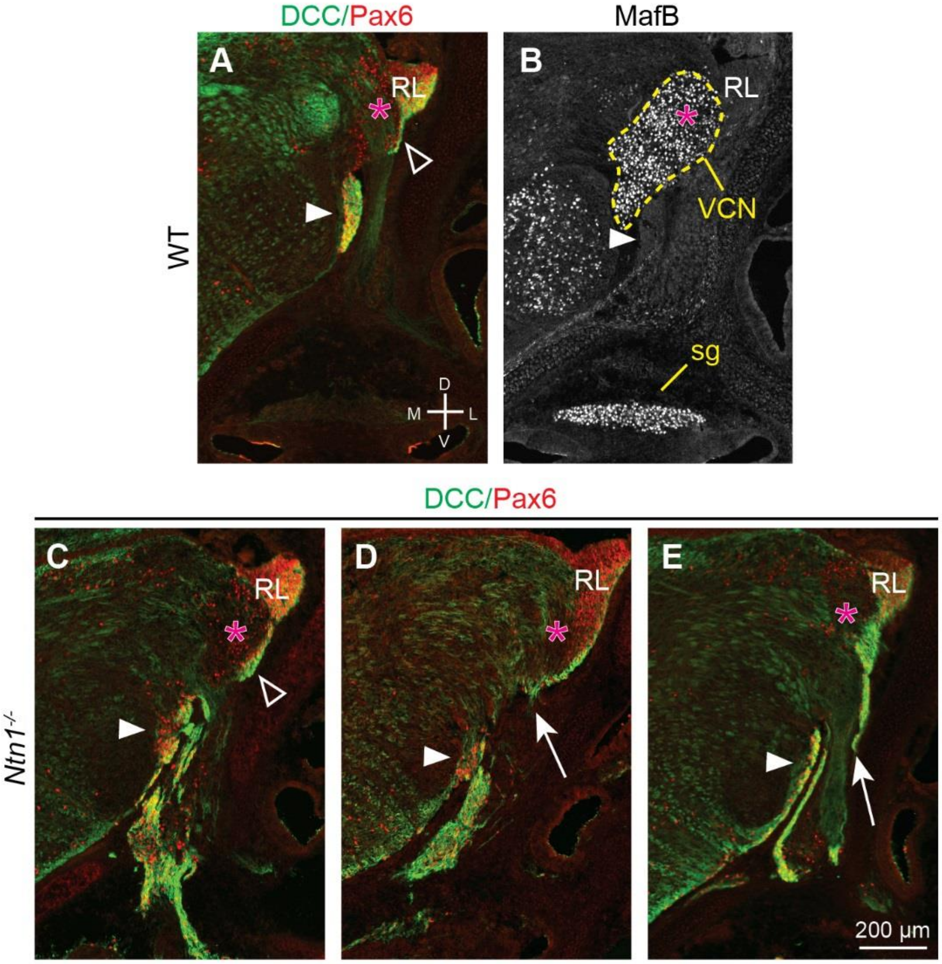

Figure S2: PNs exit the CNS independently of other populations of neurons. Related to Figure 2. (A-E) E15.5 transverse sections of the embryonic head immunostained for DCC (green, A, C-E) and Pax6 (red, A, C-E) or for MafB (B), a marker of earlier born ventral cochlear nucleus (VCN) neurons (Howell *et al.,* 2007). Comparisons between anatomically similar WT sections (A, B) reveal that the MafB+ VCN (*; outlined in B) lies ventromedial to the DCC/Pax6+ secondary rhombic lip (RL) and dorsal to the DCC/Pax6+ AES (filled arrowhead). In 5 out of the 9 *Ntn1*$^{-/-}$ embryos examined, DCC+ VCN axons appear confined to the CNS (C, hollow arrowhead) as in WT animals (A, hollow arrowhead). In the remaining 4/9 animals, VCN axons were seen projecting along the outside of the VIIIth nerve to varying degrees (D, E; arrows). However, in all cases, AES neurons exited the CNS and followed a path that was distinct from the occassional ectopic VCN axons, which departed via the more medial aspect of the VIIIth nerve. AES, anterior extramural stream; RL, secondary rhombic lip; sg, spiral ganglion; *, VCN.

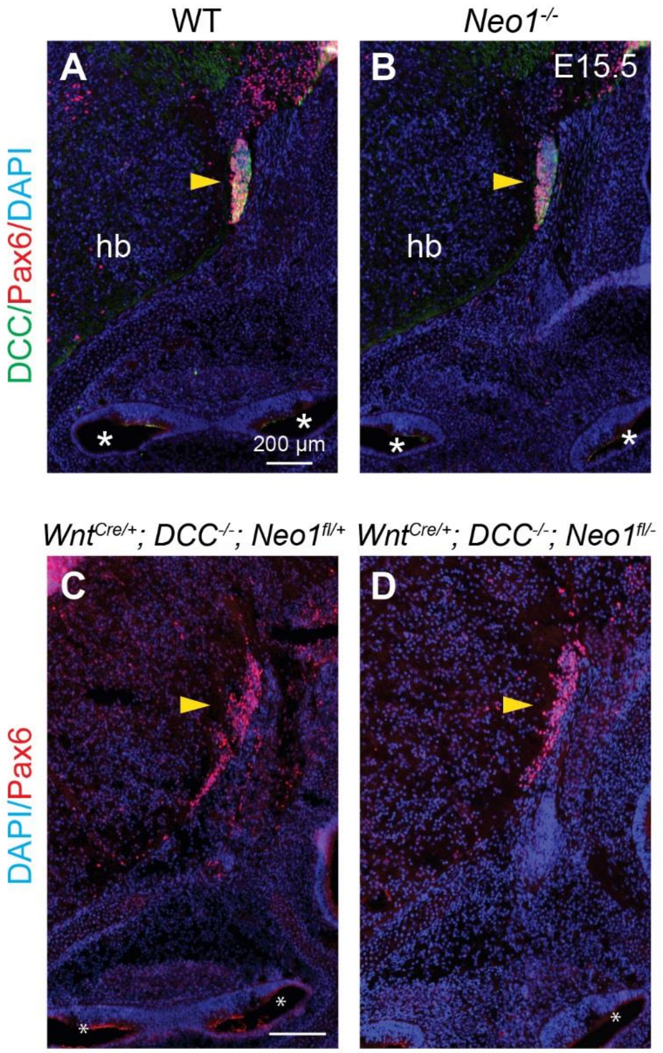

Fig. S3: PNs migrate normally in complete *Neo1* null mutants, and loss of *Neo1* selectively from the rhombic lip does not enhance CNS departure in *DCC*$^{-/-}$ animals. Related to Figure 4. (A-D) Immunostains in E15.5 receptor mutants. The AES (yellow arrowhead), indicated by Pax6 (red) and DCC (green, A-B) immunoreactivity, looks grossly normal in complete *Neo1* nulls (A, B). Loss of *Neo1* specifically in rhombic lip precursors (D) did not greatly enhance the *DCC* single mutant phenotype (C). In both cases the AES is semi-intact, and there are a smattering of neurons residing outside of the CNS. Many of these Pax6+ neurons were still located within the vestibulocochlear nerve and had not yet traveled to the cochlea proper. Hb, hindbrain; *, cochlear duct.

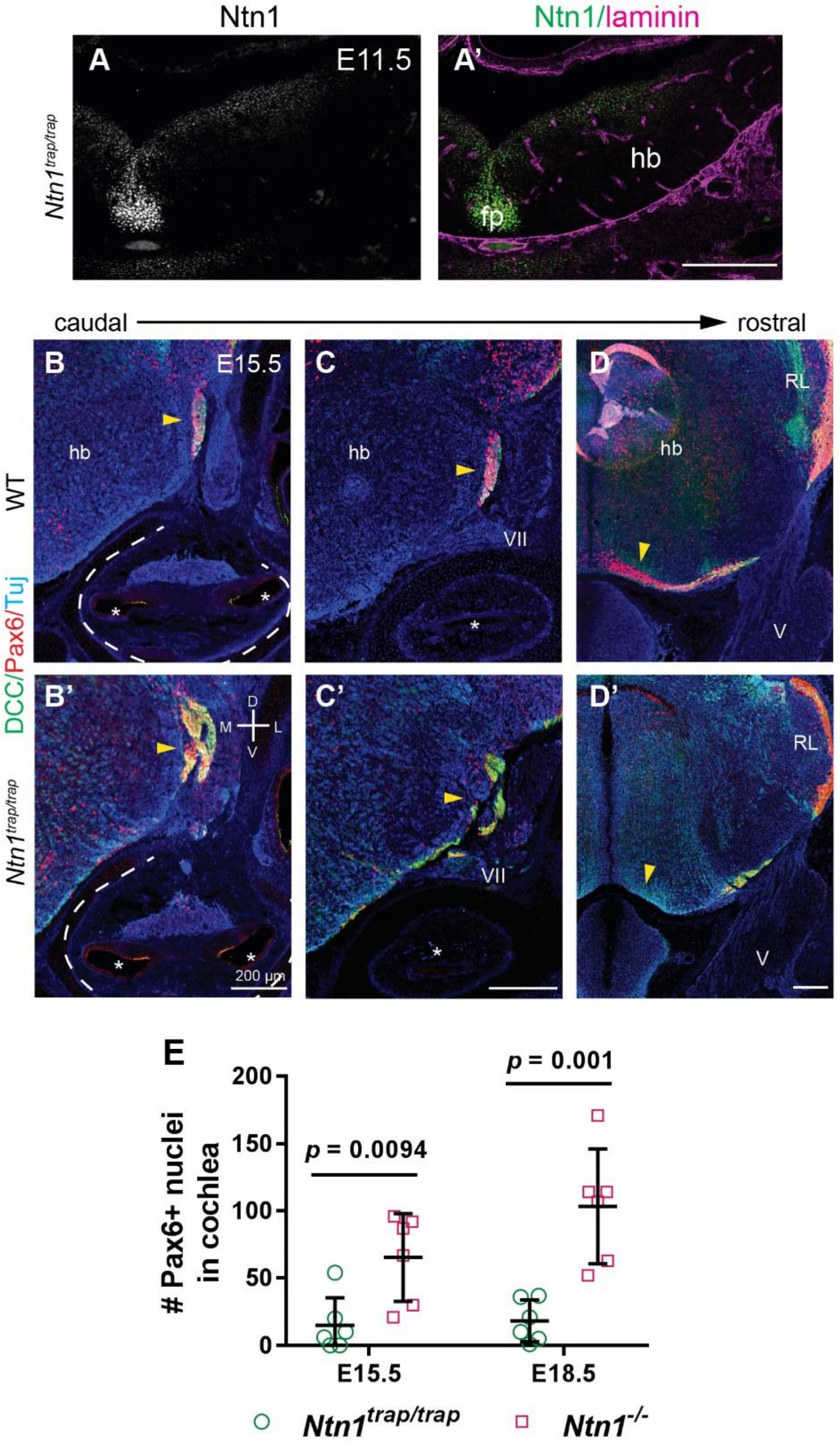
Ntn1
Ntn1/laminin
A
E11.5
A'
hb
fp
Ntn1trap/trap
caudal
rostral
B
E15.5
C
D
hb
hb
hb
RL
WT
VII
V
DCC/Pax6/Tuj
B'
C'
D'
D
M
L
V
RL
Ntn1trap/trap
VII
V
200 µm
E
p = 0.0094
p = 0.001
# Pax6+ nuclei in cochlea
200
150
100
50
0
E15.5
E18.5
Ntn1trap/trap
Ntn1-/-

Fig. S4: Fewer PNs exit the CNS in hypomorphic *Ntn1* mutants. Related to Figure 6. (A-D') Immunostains of transverse sections of *Ntn1*$^{trap/trap}$ embryonic heads. (A-A') Ntn1 (green) and laminin (magenta) immunostaining at E11.5 shows an absence of Ntn1 at the SPR and puncta reflecting trapped Ntn1 fusion protein at the floor plate and ventricular zone. (B-D') Immunostaining for Tuj (blue), Pax6 (red) and DCC (green) at E15.5 reveal ectopic PNs in hypomorphs (B'-D'). Compared to the intact AES along the sub-pial region in WT animals (B-D, yellow arrowheads), the stream of migrating PNs appears disrupted in *Ntn1*$^{trap/trap}$ animals, with some departing into the periphery at the level of the VIIIth (B') and VIIth (C') nerve roots. Many PNs get close to the midline (D'), which is rarely observed in complete nulls (see Fig. 2), but the pontine nuclei still fail to form. (E) Quantification of ectopic Pax6+ neurons in the base and middle turns of the cochlea in hypomorphic vs. complete null mice (mean ± S.D., Student's t-test). Fp, floor plate; hb, hindbrain; V, trigeminal nerve; VII, facial nerve.

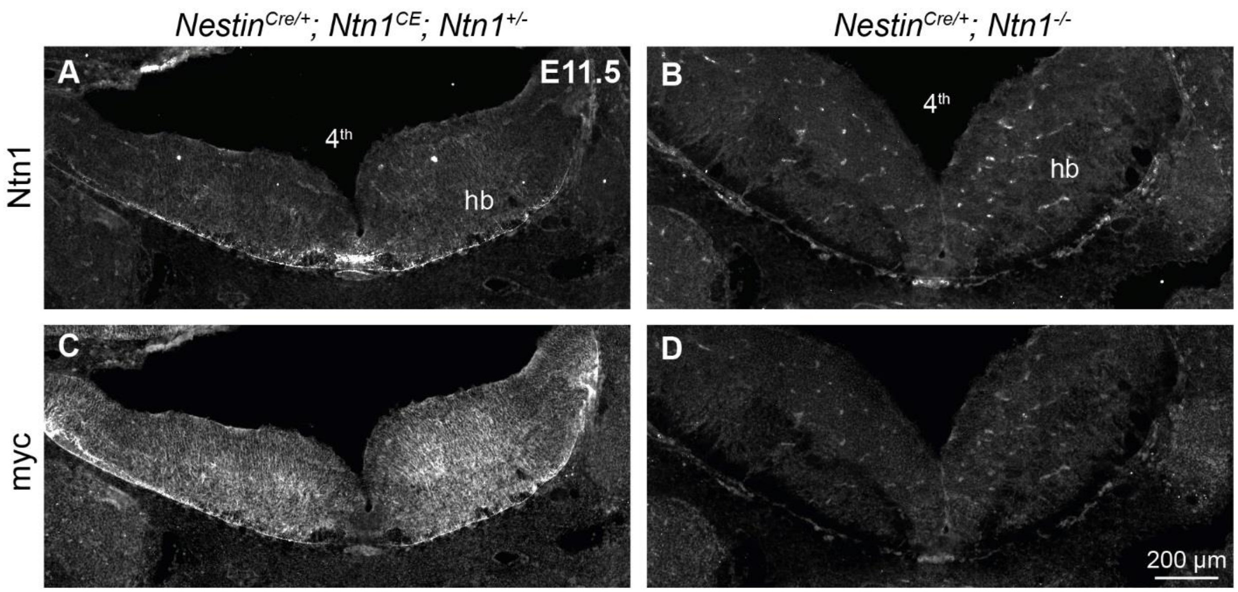

Fig. S5: Ntn1 and myc antibodies are epitope-specific. Related to Figure 7. (A-D) Immunostains of transverse sections of E11.5 embryonic heads. (A-B) Ntn1 immunostaining is eliminated in *Ntn1*$^{-/-}$ animals. (C-D) Myc immunostaining is only present in the presence of Cre and the *Ntn1* conditional expressor allele. 4th, fourth ventricle; hb, hindbrain.

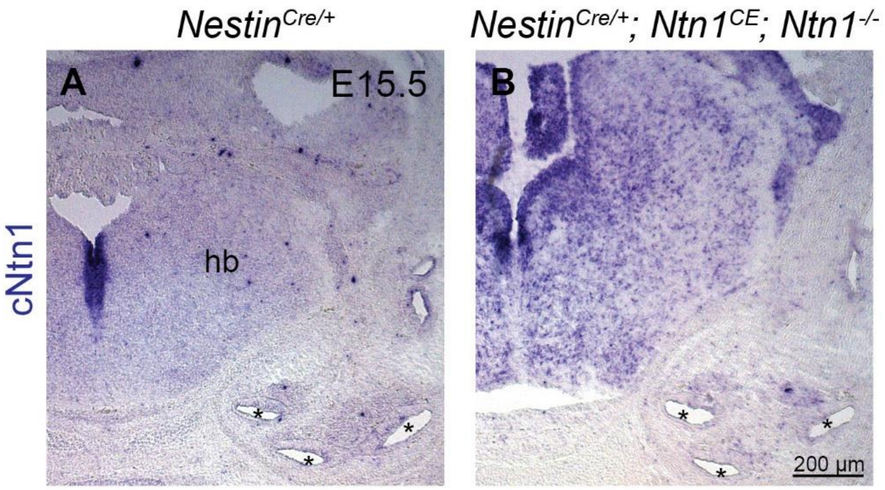

Fig. S6: *Nestin*^{Cre} drives broad *cNtn1* expression in the hindbrain. Related to Figure 7. (A-B) *In situ* hybridization for *cNtn1* shows cross-reactivity with endogenous *mNtn1* at the midline (A); *cNtn1* expression expands throughout the rest of the hindbrain in the presence of the conditional allele (B). Hb, hindbrain; *, cochlear ducts.

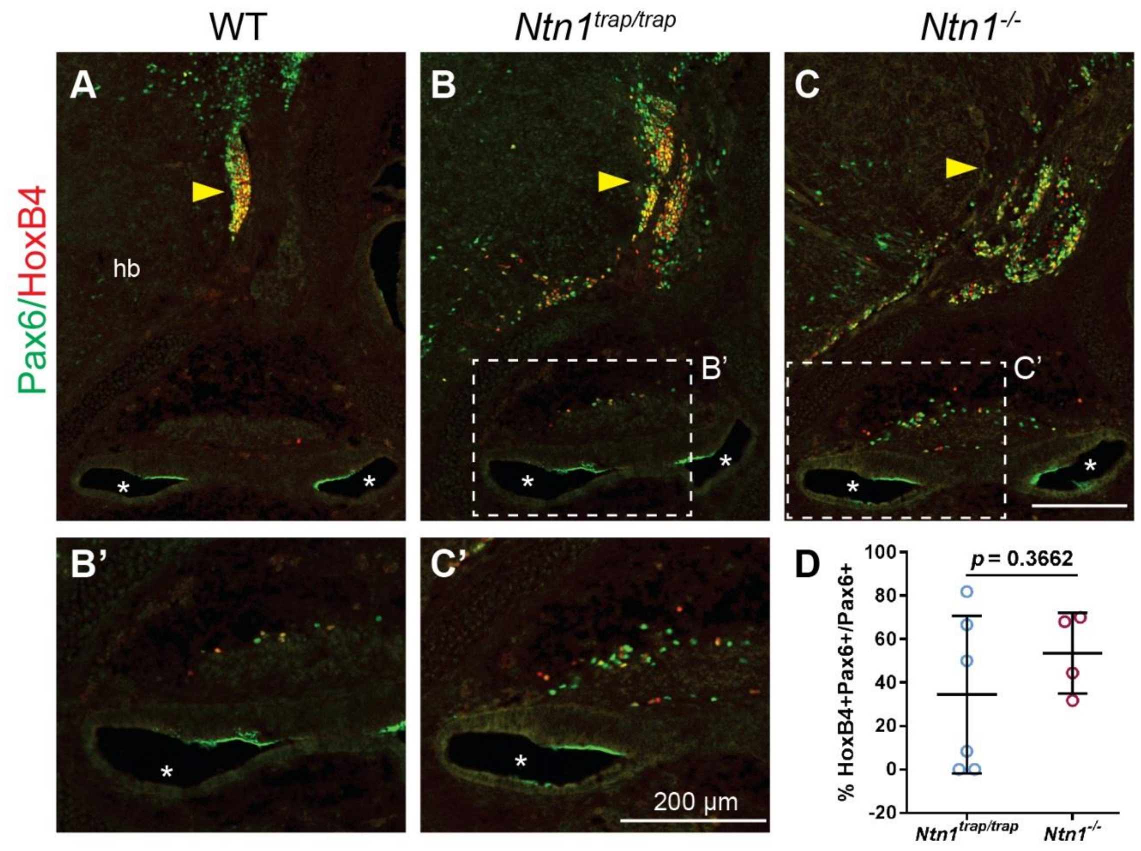

Fig. S7: PN subsets do not preferentially exit the CNS in *Ntn1* mutants. Related to Figure 4. (A-C) E15.5 transverse embryonic head sections immunostained for Pax6 (green), which labels all PNs, and HoxB4 (red), which labels subsets of PNs in a dorsoventral gradient (dorsal low, ventral high) (di Meglio et al., 2013). When compared to WT animals (A), *Ntn1* hypomorphs appear to retain this gradient of expression in remnants of the AES (B), indicating that the identity of PN subsets are preserved. We find that both HoxB4+ and HoxB4- subsets of PNs exit the CNS when Ntn1 levels are reduced (B) or eliminated (C), and they appear in the base of the cochlea at similar rates (B', C'), quantified in (D) (mean ± S.D., Student's t-test). Hb, hindbrain; *, cochlear duct.

Supplemental Experimental Information

Immunohistochemistry

Embryos were fixed in 4% paraformaldehyde (PFA)/PBS at 4°C overnight, cryoprotected in sucrose, frozen in NEG-50 (Thermo-Scientific), and sectioned at 12 to 16 µm. Sections were blocked in 3% BSA and incubated in the following primary antibodies at 4°C overnight: 1:500 goat anti-DCC (Santa Cruz), 1:750 rabbit anti-laminin (Sigma), 1:500 rabbit anti-MafB, 1:250 rat anti-myc (Santa Cruz), 1:500 goat anti-Netrin-1 (R&D), 1:400 goat anti-Neogenin (R&D), 1:400 rabbit anti-Pax6 , 1:100 mouse anti-RC2 (DSHB), 1:100 goat anti-TAG1 (R&D), and 1:1000 mouse anti-Tuj1 (Covance). For antigen retrieval, the sections were treated with boiling 10 mM sodium citrate, pH 7.0, for 20 minutes prior to blocking. Species-specific secondary antibodies conjugated to Alexa-Fluor fluorophores from Jackson ImmunoResearch or Invitrogen were used afterward.

Whole-mount brains were dissected and fixed in 4% PFA/PBS at 4°C overnight and blocked in 10% normal donkey serum (NDS) and 1% Triton-X in PBS at 4°C overnight. After incubating in primary antibody for 3 nights, the brains were incubated in HRP-conjugated secondary antibodies. Detection was performed using a DAB substrate.

In situ *hybridization*

Standard *in situ* hybridization was performed as described on 12 µm sections (Abraira et al., 2008). The *Egr2* probe was provided by Advanced Cell Diagnostics (ACD, Hayward, CA) for use with their RNAscope Fluorescent Multiplex Kit. Tissue sections were rinsed with PBS to wash off residual Neg-50 and treated with protease III (ACD) before following the manufacturer's protocol.

Transmission electron microscopy (TEM)

Embryos were collected and rinsed in 0.1 M sodium cacodylate buffer before drop-fixing in a modified Karnovsky fixative (2.5% paraformaldehyde, 5% glutaraldehyde, and 0.06% picric acid in 0.2 M cacodylate buffer) (Ito and Karnovsky, 1968) for 3-5 days at 4˚C. Whole fixed embryos were then embedded in epon resin, and ultrathin sections of 80 nm were collected on copper grids and counter-stained with Reynold's lead citrate (0.2% lead citrate).

Imaging

Images were collected on an Olympus VS120 slide scanner at 10X and 20X. Higher power images were taken with an Olympus Fluoview 1200 at 40X or a Leica SP8 confocal microscope at 25X or 40X. A 1200EX electron microscope (JEOL) equipped with a 2k CCD digital camera (AMT) captured all TEM images. Images were processed using ImageJ (NIH) and Adobe Photoshop.

Image Quantification

To quantify the number of Pax6+ nuclei in the cochlea, we counted the number of labeled cells in the base and middle turns of the cochlea in the earliest section where all three turns of the cochlea are first visible. To perform the same analyses in the VIIth nerve root, we only used sections where the nerve could be seen exiting the hindbrain to be confident of the anatomy. We counted all cells present in a 110 x 218 pixel box over this initial segment of the VIIth nerve.

The area covered by laminin was measured by centering a 561 x 386 pixel box over the BM adjacent to the AES, with the top of the box meeting the dorsal tip of the BM where it stops to permit the entry of the VIIIth nerve. After thresholding, the area covered by laminin within the box was calculated.

Ntn1 intensity was measured using confocal images at the floor plate or at the lateral edges of the hindbrain. The z-stack was summed and converted to an 8-bit image, and a 158 μm^2 circle was placed at the floor plate or at the basement membrane immediately ventral to the VIIIth nerve root. If a blood vessel was present, the circle was placed at the next available area. Each data point in the figure represents the mean intensity found over the area of the circle. All image analyses were performed using ImageJ (NIH).

Supplemental References

Ito, S., and Karnovsky, M. (1968). Formaldehyde-glutaraldehyde fixative containing trinitro compounds. J. Cell Bio. *39*, 168–169.
