# Supplementary material for: Netrin-1 Confines Rhombic Lip-Derived Neurons to the CNS
Source: Cell Rep. Author manuscript; Available in PMC 2018 Mar 30. (PMC5877811; doi:10.1016/j.celrep.2018.01.068)
Supplement: 2 [file NIHMS943889-supplement-2.pdf]

Cell Reports

Article

Netrin-1 Confines Rhombic Lip-Derived Neurons to the CNS

Graphical Abstract

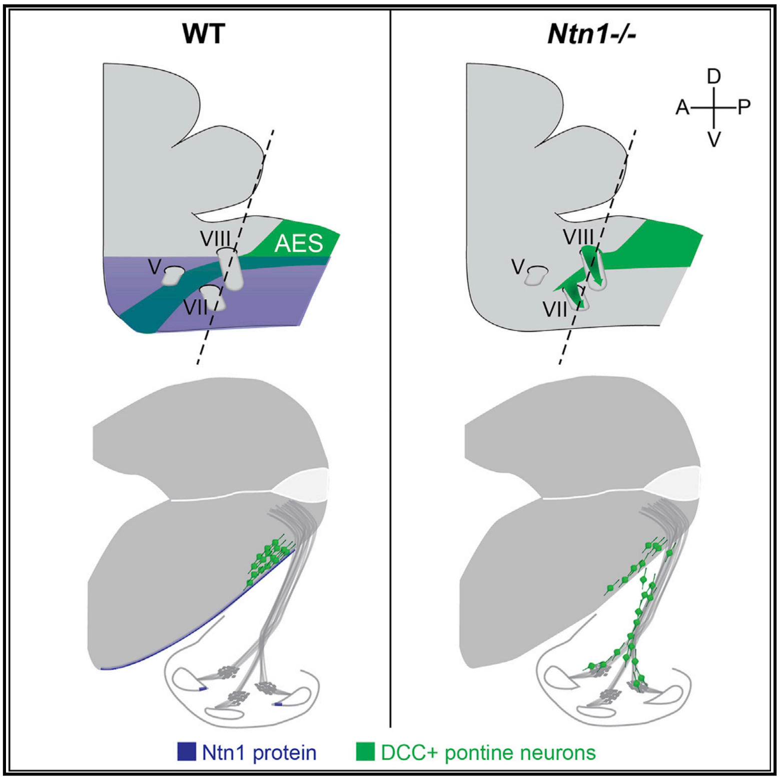

Highlights

- Ntn1 is enriched in the sub-pial region (SPR) but absent at cranial nerve roots
- Pontine neurons exit the CNS along nerves when SPR-localized Ntn1 is removed
- DCC and Neogenin act in distinct cell types to confine pontine neurons to the CNS
- Restoring Ntn1 to the SPR rescues the CNS-PNS boundary and pontine neuron migration

Authors

Andrea R. Yung, Noah R. Druckenbrod, Jean-François Cloutier, Zhuhao Wu, Marc Tessier-Lavigne, Lisa V. Goodrich

Correspondence

lisa_goodrich@hms.harvard.edu

In Brief

Yung et al. show that Ntn1 prevents pontine neurons from migrating into the periphery along cranial nerves by providing a preferred substrate in the sub-pial region. These findings introduce a local, permissive role for Ntn1 in the maintenance of the CNS-PNS boundary in the developing mouse hindbrain.

Yung et al., 2018, Cell Reports *22*, 1666–1680
February 13, 2018 © 2018 The Author(s).
https://doi.org/10.1016/j.celrep.2018.01.068

CellPress

OPEN ACCESS
CellPress

Cell Reports
Article

Netrin-1 Confines Rhombic Lip-Derived Neurons to the CNS

Andrea R. Yung,[1] Noah R. Druckenbrod,[1] Jean-François Cloutier,[2] Zhuhao Wu,[3] Marc Tessier-Lavigne,[2,5] and Lisa V. Goodrich[1,4,*]
[1]Department of Neurobiology, Harvard Medical School, Boston, MA, USA
[2]Department of Neurology & Neurosurgery, Montreal Neurological Institute, McGill University, Montreal, QC, Canada
[3]Laboratory of Brain Development & Repair, The Rockefeller University, New York, NY 10065, USA
[4]Lead Contact
[5]Present address: Department of Biology, Stanford University, Stanford, CA 94306, USA
*Correspondence: lisa_goodrich@hms.harvard.edu
https://doi.org/10.1016/j.celrep.2018.01.068

SUMMARY

During brainstem development, newborn neurons originating from the rhombic lip embark on exceptionally long migrations to generate nuclei important for audition, movement, and respiration. Along the way, this highly motile population passes several cranial nerves yet remains confined to the CNS. We found that Ntn1 accumulates beneath the pial surface separating the CNS from the PNS, with gaps at nerve entry sites. In mice null for Ntn1 or its receptor DCC, hindbrain neurons enter cranial nerves and migrate into the periphery. CNS neurons also escape when Ntn1 is selectively lost from the sub-pial region (SPR), and conversely, expression of Ntn1 throughout the mutant hindbrain can prevent their departure. These findings identify a permissive role for Ntn1 in maintaining the CNS-PNS boundary. We propose that Ntn1 confines rhombic lip-derived neurons by providing a preferred substrate for tangentially migrating neurons in the SPR, preventing their entry into nerve roots.

INTRODUCTION

A basic organizing principle of the nervous system is the segregation of the peripheral nervous system (PNS) and CNS, which are anatomically and functionally distinct yet linked by nerves. This is particularly apparent in the vertebrate brainstem, which houses ten cranial nerves as well as a constellation of nuclei that govern functions critical to life, from motor coordination to auditory processing (Farago et al., 2006; Wang et al., 2005). Many of these nuclei are composed of neurons originating from the rhombic lip, a transient strip of proliferating neuroepithelium lining the fourth ventricle during development (Ray and Dymecki, 2009). The formation of hindbrain nuclei, therefore, depends on the successful tangential migration of newborn neurons from the rhombic lip to their final destinations. This route is unusually long and complex, especially since the surface of the hindbrain is broken by multiple cranial nerve roots that the rhombic lip derivatives must ignore. Although several guidance cues play critical roles sculpting the trajectory of tangentially migrating neurons *in vivo* (reviewed in Kratochwil et al., 2017), nothing is known about the molecular mechanisms that confine these neurons to the CNS, despite opportunities to deviate into the periphery.

Pontine neurons (PNs) traverse one of the longest migratory routes in the hindbrain, ultimately settling at the midline to supply excitatory mossy fiber input to the cerebellum (Kratochwil et al., 2017). PNs originate from the rhombic lip in rhombomeres (r)6–r8 from embryonic day (E)12.5 to E16.5. They extend long leading processes (Ono and Kawamura, 1990; Yee et al., 1999) as they migrate beneath the pial surface, maneuvering between the trigeminal (Vth), facial (VIIth), and vestibulocochlear (VIIIth) nerve roots and arriving at the ventral midline of r3–r4 several days later (Nichols and Bruce, 2006) (Figure 1A). This navigation depends on the activity of several guidance cues, including Slits, which are secreted by the facial motor nucleus to prevent premature ventral migration (Geisen et al., 2008), and meninges-derived chemokine SDF-1, which keeps PNs from migrating into the neuroepithelium (Zhu et al., 2009). What prevents PNs from escaping in the opposite direction, into the periphery, is unknown.

One of the first guidance cues implicated in PN migration is the classic chemoattractant Netrin-1 (Ntn1). PNs are highly sensitive to Ntn1 and can migrate toward a source of Ntn1 over unusually long distances *in vitro* (Yee et al., 1999); *in vivo*, the pontine nuclei are missing in mice severely hypomorphic for Ntn1 (Serafini et al., 1996; Yee et al., 1999). These data were originally interpreted to indicate that a floor-plate-derived gradient of Ntn1 guides PNs to the midline during the final leg of their migration (Zelina et al., 2014), much as Ntn1 was proposed to guide commissural axon growth in the developing spinal cord (Kennedy et al., 2006; Serafini et al., 1996). However, Ntn1 is also expressed in the ventricular zone (Kennedy et al., 1994; Serafini et al., 1996), and this source is required for proper commissure formation (Charron et al., 2003). The protein itself is deposited in the sub-pial region (SPR) adjacent to the basement membrane (BM) surrounding the neural tube (Kennedy et al., 2006; MacLennan et al., 1997; Varadarajan et al., 2017). Recent genetic studies have underscored the importance of SPR-localized Ntn1 for commissural axon guidance (Dominici et al., 2017; Varadarajan et al., 2017; Yamauchi et al., 2017), consistent

This is an open access article under the CC BY license (http://creativecommons.org/licenses/by/4.0/).

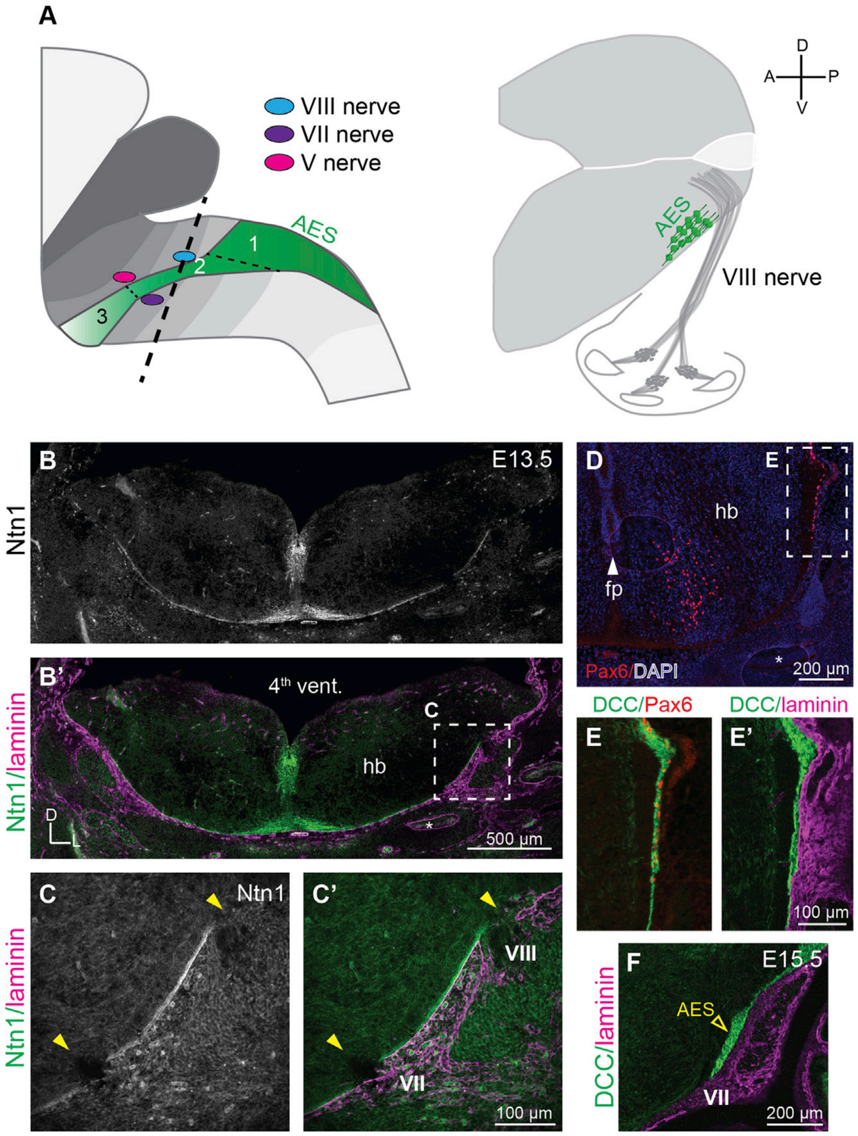

Figure 1. Ntn1 Protein Is Enriched in the SPR in the Developing Hindbrain

(A) Schematic depicting the three phases of PN migration (green) across multiple rhombomere segments (shaded in gray) and a view of the AES in an E15.5 transverse section. Thick dashed line indicates plane of section. D, dorsal; A, anterior; P, posterior; V, ventral.

(B–F) Immunostains of transverse embryonic head sections. At E13.5, low-power (B and B') and high-power (C and C') images show strong Ntn1 staining at the FP, on crossing commissural axons at the midline, and in the sub-pial region (SPR) adjacent to the laminin-positive pial basement membrane (magenta). Curiously, Ntn1 appears to be absent from nerve roots (yellow arrowheads). Low (D) and high (E-E') magnification images show ventrally migrating pontine neurons in the SPR, even as they avoid cranial nerve roots later in their migration (F; E15.5). Pontine neurons express Pax6 (red, D and E) and DCC (green, E–F).

AES, anterior extramural stream; fp, floor plate; hb, hindbrain; 4th vent., fourth ventricle; VII, facial nerve; VIII, vestibulocochlear nerve.

with documented functions for Ntn1 in the BM of other tissues (Liu et al., 2004; Srinivasan et al., 2003; Yebra et al., 2003). This suggests that additional roles for locally produced Ntn1 in the developing nervous system likely remain to be found, particularly in cases such as PN migration, where neurons migrate along the pial surface rather than through the neuroepithelium. Indeed, the complete range of Ntn1's effects on PN migration is still unclear, due both to the hypomorphic nature of the original allele and to the lack of information about the ultimate fate of PNs.

Here, we show that Ntn1 functions as a permissive cue to confine PNs to the CNS. We propose that Ntn1 in the sub-pial region provides a preferred corridor for migrating rhombic lip-derived neurons, allowing them to distinguish the appropriate migratory substrate and avoid opportunities to migrate instead into the periphery. These findings introduce another local function for Ntn1 and establish an additional molecular explanation for how CNS-PNS segregation is achieved in the brainstem, a hub for CNS-PNS interactions that are vital for life.

RESULTS

Ntn1 Protein Is Enriched in the SPR in the Developing Hindbrain

Migrating PNs follow a stereotyped pathway to the ventral midline that can be divided into three phases: (1) a short ventral migration marking the departure from the rhombic lip; (2) a relatively straight rostral migration between and past the vestibulocochlear (VIIIth), facial (VIIth), and trigeminal (Vth) nerves, respectively; and (3) a final ventral turn before resting at the midline (Figure 1A) (Geisen et al., 2008; Nichols and Bruce, 2006). The entire migration takes place in the space beneath the pia, which we call the sub-pial region (SPR). While the molecules that define many aspects of this complex migratory route have been identified, the cues that instruct PNs to stay in the SPR and avoid cranial nerve roots remain unknown. During the peak of migration at E15.5, PNs traverse hundreds of microns as they move past the cranial nerves. Throughout this journey, PNs express the Ntn1 receptor Deleted in Colorectal Carcinoma (DCC) and are exposed to Ntn1 produced in the floor plate (FP) and ventricular zone (Yee et al., 1999). When *Ntn1* levels are reduced, rare spinal cord interneuron axons can be found in dorsal root ganglia (Laumonnerie et al., 2014), hinting at a role in setting the CNS-PNS boundary. We therefore hypothesized that Ntn1 not only guides PNs to the midline but also keeps them contained within the CNS.

Since Ntn1 is a potent secreted cue, we examined the localization of Ntn1 relative to its sources in the hindbrain at the onset of PN migration at E13.5 (de Diego et al., 2002; Yee et al., 1999). Immunostaining revealed that Ntn1 protein is widely distributed (Figures 1B and 1B'; n = 2 animals), accumulating in the FP, on commissural axons, and in the SPR in the vicinity of the laminin-positive pial BM, as described previously (Dominici et al., 2017; Kennedy et al., 2006; MacLennan et al., 1997; Varadarajan et al., 2017). Notably, Ntn1 is absent from nerve roots, where cranial nerves project into or out of the CNS via gaps in the pial BM at stereotyped locations (Figures 1C and 1C'; n = 2 animals). Thus, Ntn1 protein is present where PNs migrate, but not at sites they avoid.

To determine whether migrating PNs might encounter and respond to Ntn1 in the SPR, we stained for PN markers Pax6 and DCC at E13.5, when PNs have begun exiting the rhombic lip, and at E15.5, when they are passing by cranial nerves. At E13.5, Pax6+/DCC+ cells cluster beneath the pial BM (Figures 1D–1E'; n = 4 animals) and maintain this position as they navigate near the facial (VII) and vestibulocochlear (VIII) nerves at E15.5 (Figure 1F). Since Ntn1 is enriched in the SPR, migrating DCC+ PNs likely encounter Ntn1 from early on. Given the conspicuous absence of Ntn1 at nerve roots—which migrating PNs avoid—this pattern of distribution suggests that Ntn1 may contribute to the confinement of tangentially migrating neurons in the hindbrain by providing an attractive substrate.

Loss of *Ntn1* Causes PNs to Exit the Hindbrain and Enter the Periphery

Earlier analyses of hypomorphic *Ntn1* animals (*Ntn1$^{trap/trap}$*) suggested that Ntn1 mediates the final ventral migration to the midline, as PNs complete most of the first and second phases of their migration (Zelina et al., 2014). However, phenotypic analyses of *Ntn1* null animals (*Ntn1$^{-/-}$*) showed that residual Ntn1 in hypomorphs masks the full extent of its role in guidance (Bin et al., 2015; Yung et al., 2015). We posited that complete loss of Ntn1 might reveal additional functions earlier in migration, particularly since PNs are exposed to Ntn1 far before their final ventral turn.

To visualize PN distribution, we collected transverse sections of embryonic *Ntn1$^{-/-}$* heads spanning the anterior extramural stream (AES) through which PNs travel. In E15.5 control animals (n = 4), the AES is identifiable as a dense stripe of Pax6+ nuclei and DCC+ processes traveling beneath the pial surface (Figures 2B–2D), giving rise to the pontine nuclei at the midline, which first appear at late E14.5 (Nichols and Bruce, 2006). In contrast, in *Ntn1$^{-/-}$* animals (n = 6), the AES was missing, and there were ectopic streams of Pax6+ and DCC+ neurons immediately ventral to the stereotyped location of the AES, as if the PNs had been diverted into the periphery (Figures 2B' and 2C'). Ectopic Pax6+ nuclei and a few DCC+ processes were rarely found in the trigeminal (Vth) ganglion (Figure 2D'). However, the facial and vestibulocochlear nerves contained similar numbers of ectopic nuclei (Figure 2E; n = 6 nerves each). We focused on the vestibulocochlear (VIIIth) nerve as a site of exit, as it is the first nerve root that migrating PNs pass and because the cochlea is an enclosed, easily recognizable landmark. These findings suggest that, in addition to mediating the final ventral turn to the midline, Ntn1 acts earlier to prevent PNs from migrating along cranial nerves and into the periphery.

To confirm the origin and identity of these neurons, we genetically labeled PNs by providing tamoxifen to E13.5 *Atoh1^{CreERT2};Ai14* mice crossed onto the *Ntn1* null background. tdTomato+ cells also expressed both Pax6 and DCC (Figures 2G–2H'''; n = 3 animals), demonstrating that the ectopic neurons in the cochlea derive from the rhombic lip. Likewise, in *Ntn1* mutants, Pax6+ neurons first reach the cochlea at E13.5 and increase in number steadily up until E15.5 (Figure 2F; n = 6 cochleae per time point), matching the timing of PN production and migration. These neurons accumulate mostly in the base and middle turns of the cochlea, which lie closest to the hindbrain. Taken together, these data demonstrate that the Pax6+ neurons invading *Ntn1$^{-/-}$* cochleae are *bona fide* PNs.

Despite their ectopic location, the misrouted PNs survived and differentiated within the cochlea. At E18.5, 103.5 ± 42.6 (mean ± SD) Pax6+ PNs were present in the cochlea, but instead of integrating into the spiral ganglion, the PNs, which express low levels of Gata3, formed a rind around the Gata3-high spiral ganglion neurons (SGNs; Figures 3A–3C'; n = 3 *Ntn1$^{-/-}$*). The gross organization of the spiral ganglion was remarkably normal, as visualized by crossing a *MafB^{GFP}* allele (Moriguchi et al., 2006) into the *Ntn1$^{-/-}$* background. As in control embryos, GFP+ SGNs extended orderly bundles of radial fibers toward the hair cells in mutants (Figures 3D and 3F; n = 3 *Ntn1$^{-/-}$*), beneath an overlying swath of processes from GFP−/Tuj+ PNs (Figures 3E and 3G). Since *Ntn1* mutants die at birth, we could not examine the fate of ectopic PNs in adults. Nonetheless, these data show that PNs thrive in the cochlea but stay segregated from the SGNs.

Multiple Populations of Neurons Escape the CNS in *Ntn1* Mutants

In addition to PNs, many other rhombic lip derivatives migrate through the SPR, including neurons of the cochlear nucleus, inferior olive, and external cuneate nucleus (Machold and Fishell, 2005; Nichols and Bruce, 2006; Wang et al., 2005). As these neurons also respond to Ntn1 (Alcántara et al., 2000; Bloch-Gallego et al., 1999; Causeret et al., 2002; Howell et al., 2007; Riyadh et al., 2014; Marcos et al., 2009), we wondered if Ntn1's role in confinement extends to other rhombic lip derivatives. Since *Ntn1* is expressed in the neural tube as early as E9.5 (Kennedy et al., 1994; Serafini et al., 1994; Yee et al., 1999), we examined the trajectory of some of the earliest born rhombic lip-derived neurons in the CNS by injecting tamoxifen at E9.5 into *Atoh1^{CreERT2};Ai14* mice crossed to the *Ntn1* null background. As expected, many commissural neurons were labeled, shown by the presence of tdTomato+ processes crossing the midline at E11.5 (Figures S1A'–S1A'''; n = 2 controls). In *Ntn1$^{-/-}$* animals, tdTomato+ neurons resided in more dorsal locations, and the commissure failed to form. Additionally, many labeled cell bodies and processes were found outside the hindbrain near and along cranial nerve roots (Figures S1B'–S1B'''; n = 3 mutants). As in older animals, the tdTomato+ cells escaped through the Vth, VIIth, and VIIIth nerves, raising the possibility that later born neurons such as PNs depend on these existing ectopic tracts to exit the CNS. However, we found that, within the VIIIth nerve, PNs were not physically associated with any other ectopic DCC+ axons and were consistently present independent of other

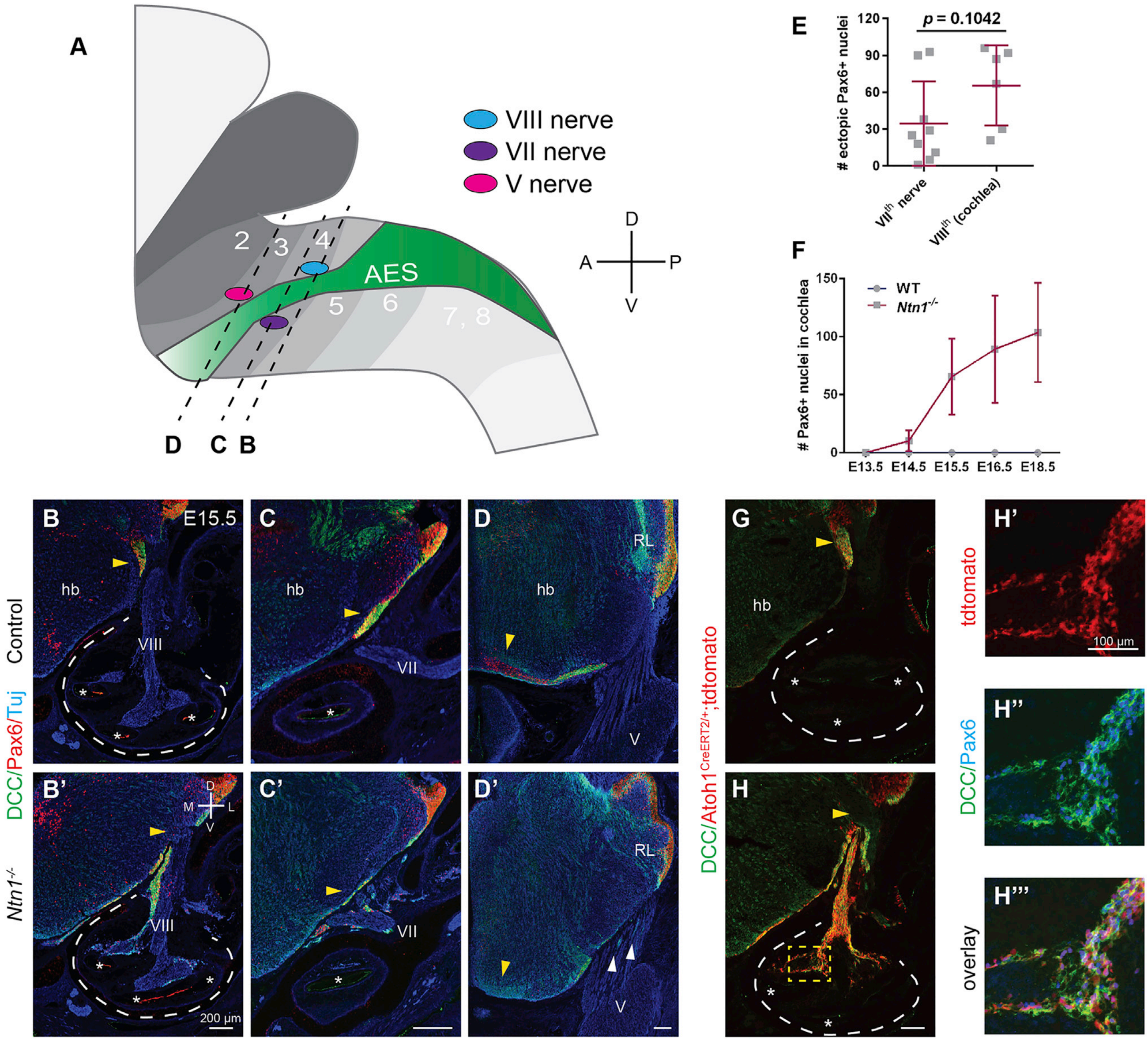

Figure 2. PNs Exit the CNS along Cranial Nerves in the Absence of *Ntn1*

(A) Schematic of the PN migratory route (anterior extramural stream, green) across multiple rhombomeres (numbered, shaded in gray) and relative to cranial nerve roots.

(B–D') E15.5 transverse head sections immunostained for DCC and Pax6 to label migrating PNs (yellow arrowheads), which normally travel rostrally beneath the pial surface toward the midline, shown at three rostro-caudal levels (B, C, and D), as indicated by the dashed lines in (A). A mix of WT and $Ntn1^{+/-}$ tissues are shown as controls. In $Ntn1^{-/-}$ animals, the AES is missing. PNs, instead, diverge into the VIIIth (B') and VIIth (C') nerves. Rare ectopic processes are present in the Vth nerve (D', white arrowheads).

(E and F) Quantification of the number of Pax6+ neurons in the VIIth and VIIIth nerves (E) (mean ± SD, Student's t test) and in the base and middle turns of control and $Ntn1^{-/-}$ cochlear sections over time (F) (mean ± SD).

(G–H''') Low- (G and H) and high-power (H'–H''') images of fate-mapped PNs in the cochlea which were labeled with tdTomato (G, H, H'), DCC (G, H, H''), and Pax6 (H'') in $Atoh1^{CreERT2/+}$; *Ai14*; $Ntn1^{+/-}$ (G) and $Ntn1^{-/-}$ (H-H''') embryos exposed to tamoxifen at E13.5. A merged image is shown in (H''').

Dotted lines indicate the cochlea; roman numerals indicate cranial nerves. hb, hindbrain. Asterisks indicate cochlear duct. All scale bars indicate 200 µm unless otherwise noted.

See also Figures S1 and S2.

ectopic projections, such as those from the earlier born neurons in the ventral cochlear nucleus (Figure S2). These observations argue that the departure of PNs is not secondary to earlier phenotypes. Thus, multiple populations of neurons exit the CNS in the absence of *Ntn1*, indicating that Ntn1 plays a general role in establishing the CNS-PNS boundary.

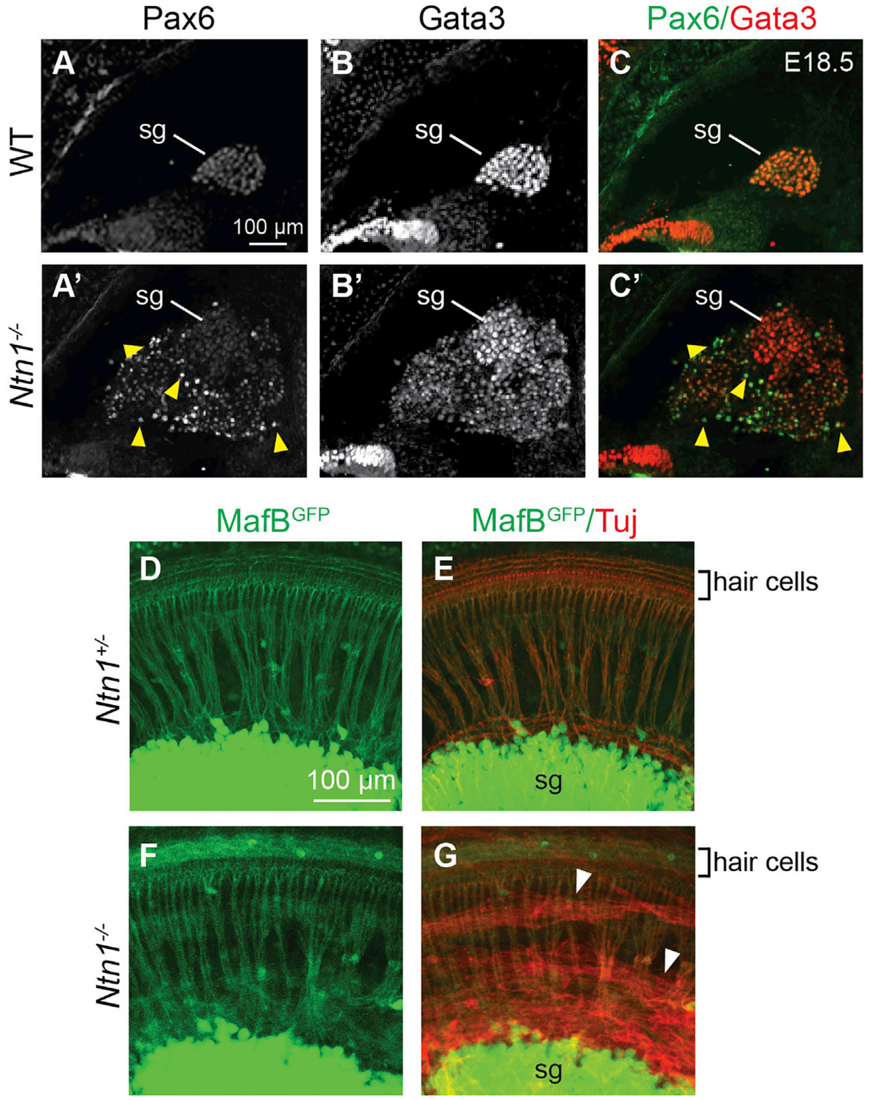

Figure 3. Ectopic Neurons Do Not Integrate into the Spiral Ganglion

(A–C') E18.5 transverse sections of the base of the cochlea immunostained for Pax6 (A and A') and Gata3 (B and B'). Only mutant cochleae contain Pax6+ neurons (yellow arrowheads), which form a rind around SGNs that express higher levels of Gata3 (C').

(D–G) Whole-mount immunostains of E18.5 cochleae from control and *Ntn1*$^{-/-}$ embryos also harboring a *MafB*^{GFP} allele, which is expressed in SGNs. GFP+ SGN processes (green, D–G) form bundles extending radially to hair cells in both *Ntn1*$^{+/-}$ controls (D and E) and mutant mice (F and G). In addition, *Ntn1*$^{-/-}$ cochleae contain Tuj-positive PNs (red, E and G) that extend MafB^{GFP} -negative GFP-processes longitudinally over the SGNs and their radial fibers (G; white arrowheads). Merged images shown in (E) and (G). sg, spiral ganglion.

DCC Is Also Required for PN Confinement

Since PNs express DCC and fail to reach the midline in *DCC*$^{-/-}$ animals (Fazeli et al., 1997; Yee et al., 1999), we hypothesized that Ntn1-DCC signaling underlies their confinement. Consistent with this idea, *DCC*$^{-/-}$ animals contain ectopic neurons in the cochlea that were assumed to be displaced SGNs (Kim et al., 2016). We found not only that ectopic neurons in *DCC*$^{-/-}$ cochlea express Pax6, indicative of PN identity instead, but also that Pax6+ neurons are also present elsewhere in the periphery, such as in the VIIth and VIIIth nerves, demonstrating that DCC enables Ntn1-mediated confinement. However, there were not as many neurons in *DCC*$^{-/-}$ cochleae as in *Ntn1* mutants (Figure 4A; n $\geq$ 6 cochlea per genotype), hinting that another receptor also contributes.

Ntn1 signals through many receptors, including Unc5 family members, Down syndrome cell adhesion molecule (DSCAM), integrins, and the DCC ortholog Neogenin (reviewed in Lai Wing Sun et al., 2011). We predicted that Neogenin might influence Ntn1-mediated confinement, since DCC and Neogenin collaborate to mediate midline crossing in commissural neurons (Xu et al., 2014). As in previous reports (Brugeaud et al., 2014; Fitzgerald et al., 2007; van den Heuvel et al., 2013), we observed low levels of Neogenin throughout the E15.5 hindbrain (Figures 4B and 4B'; n = 2 *Ntn1*$^{+/-}$), with stronger expression in the surrounding mesenchyme and SGNs. We did not detect Neogenin in migrating PNs and found no obvious qualitative differences in the size or location of the pontine nuclei or the trajectory of the AES in either *Neo1* hypomorphs (*Neo1*$^{Gt/Gt}$; Bae et al., 2009; Leighton et al., 2001) (Figures 4D–4G; n = 3 *Neo1*$^{Gt/Gt}$) or null animals (Kam et al., 2016) (Figures S3A and S3B; n = 3 *Neo1*$^{-/-}$). Ectopic PNs in the cochleae also did not express Neogenin (Figures 4C and 4C'; n = 3 *Ntn1*$^{-/-}$), suggesting that Neogenin is not required for PN migration or confinement. Nonetheless, increasing numbers of Pax6+ neurons accumulated in the cochlea, as more copies of *Neo1* were lost in the *DCC* null background (Figure 4A). Thus, Neogenin affects Ntn1-mediated PN confinement when DCC is absent, but not when DCC is present.

Neogenin Functions Non-Cell-Autonomously in PN Migration

A redundant function for Neogenin within PNs would provide the simplest explanation for why *DCC*$^{-/-}$*; Neo1*$^{Gt/Gt}$ double mutants, but not *DCC*$^{-/-}$ or *Neo1*$^{Gt/Gt}$ single mutants, more closely mimic the phenotype in *Ntn1*$^{-/-}$ animals. To test whether PNs upregulate Neogenin in the absence of DCC, we crossed the *Neo1*$^{Gt/+}$ allele into the *DCC*$^{-/-}$ background. This allowed us to assay β-galactosidase activity as a proxy for *Neo1* expression, which is more sensitive than immunostaining. As expected,

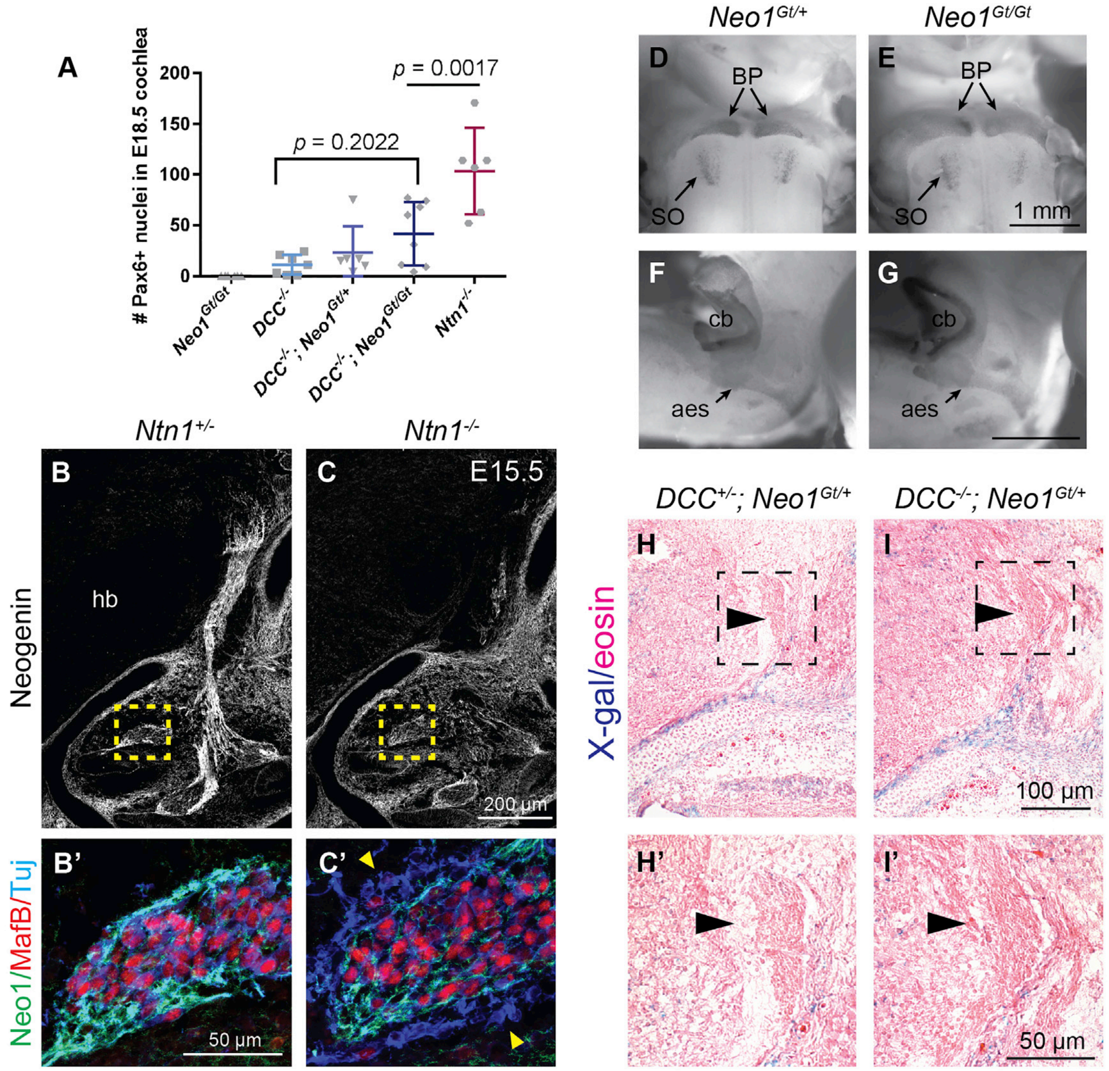

Figure 4. *DCC$^{-/-}$; Neo1$^{Gt/Gt}$* Double Mutants Phenocopy *Ntn1$^{-/-}$* Mutants, but Neogenin Acts Non-Cell-Autonomously

(A) The number of Pax6+ PNs found in the base and middle turns of the cochlea in E18.5 *DCC* and *Neo1* gene trap (*Neo1^{Gt}*) single and double mutants (mean ± SD, $p < 0.0001$, $F = 14.33$; $DF = 28$; one-way ANOVA, Tukey's multiple comparisons test).

(B–C') Immunostained E15.5 transverse sections show that Neogenin is expressed strongly in MafB+ SGNs and in the surrounding mesenchyme in control (B) and *Ntn1$^{-/-}$* (C) tissue. High-power images of the boxed areas show that MafB+ SGNs normally express Neogenin (B'), but ectopic MafB- PNs in the ear do not (C', yellow arrowheads).

(D–G) Ventral (D and E) and sagittal (F and G) views of heterozygous (D and F) and homozygous (E and G) *Neo1* E15.5 brains immunostained for Pax6. Rostral is up in (D) and (E) and to the right in (F) and (G).

(H–I') X-gal reactions (blue) in eosin-stained tissue from E15.5 control and DCC$^{-/-}$ embryos carrying the *Neo1^{Gt}* allele, which drives expression of β-galactosidase in Neogenin+ cells. No signal is detected in the AES (black arrowheads) in *DCC$^{+/-}$; Neo1$^{Gt/+}$* (H, H') or *DCC$^{-/-}$; Neo1$^{Gt/+}$* (I and I') animals, shown at low (H and I) and high (H' and I') magnification.

aes, anterior extramural stream; BP, basilar pons; cb, cerebellum; hb, hindbrain; SO, superior olive.

See also Figures S3 and S7.

β-galactosidase reaction product was present in SGNs and the surrounding mesenchyme at E15.5, but not in the AES in either control or mutants (Figures 4H–4I'; n = 3 control, 4 *DCC$^{-/-}$*). Thus, Neogenin is unlikely to compensate for DCC in migrating PNs.

To be sure that early or low levels of Neogenin in rhombic lip-derived neurons do not explain the stronger phenotype in *DCC$^{-/-}$; Neo1$^{Gt/Gt}$* double mutants, we used *Wnt1^{Cre}* and a floxed *Neo1* allele (Kam et al., 2016) to remove Neogenin from *Wnt1*+ rhombic lip precursors in a *DCC* null background. Deletion of *Neo1* from early PNs did not enhance the *DCC* phenotype (Figures S3C and S3D; n = 3 conditional mutants), making it highly unlikely that Neogenin and DCC function redundantly in PNs. Additionally, though more PNs migrated all the

OPEN
ACCESS
CellPress

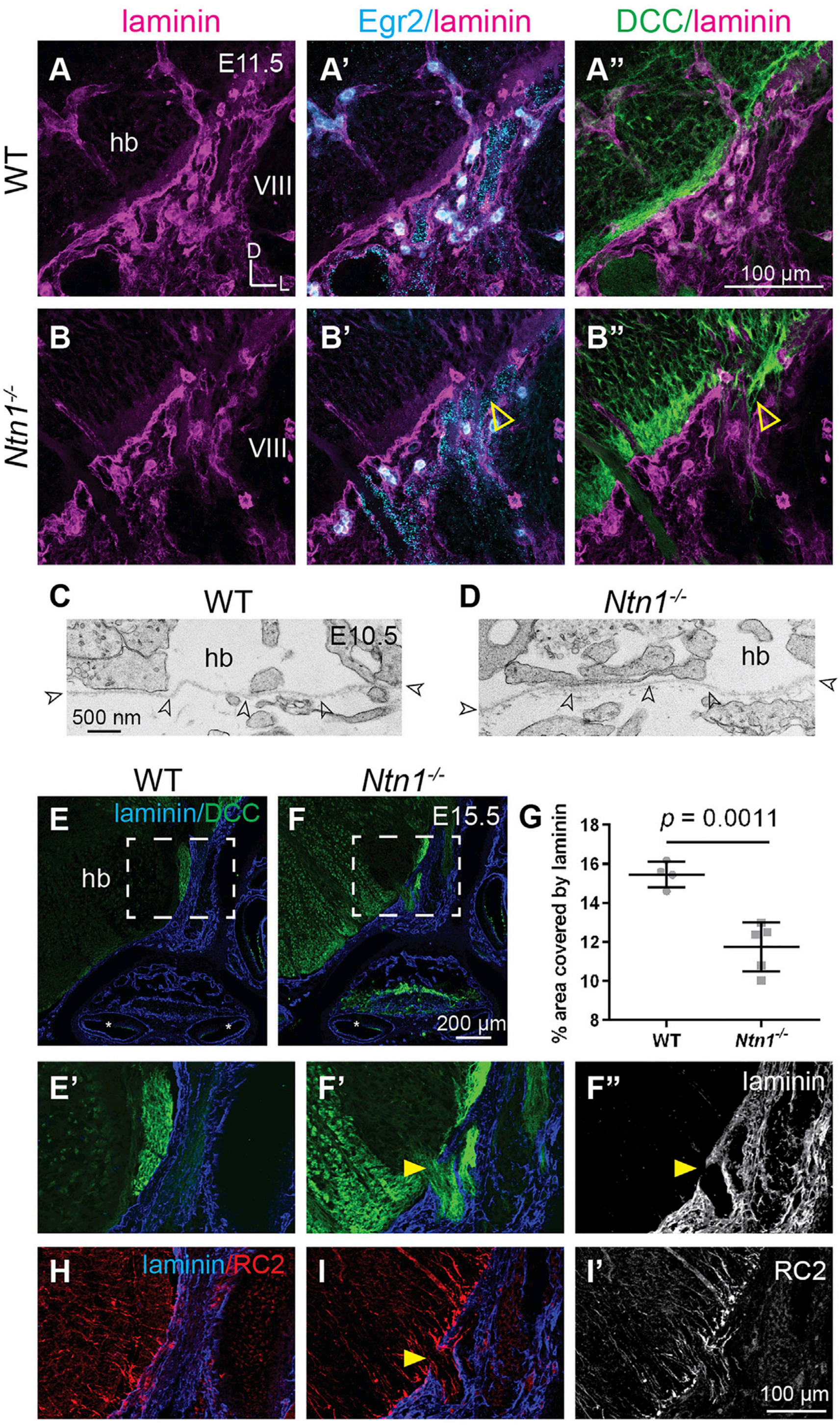

Figure 5. Neurons Exit the CNS Independent of Defects in BCCs, Radial Glial Endfeet, and the Basement Membrane

(A–B'') High-power images of E11.5 transverse head sections near the VIIIth nerve root show that Egr2+ BCCs (blue) are present at gaps in laminin (magenta) in both WT (A–A'') and *Ntn1*$^{-/-}$ (B–B'') animals. In *Ntn1*$^{-/-}$ mutants, ectopic DCC+ processes exit the CNS despite the presence of BCCs (hollow yellow arrowheads).

(C and D) TEM images of the basement membrane (BM, hollow black arrowheads) surrounding the WT (C) and *Ntn1*$^{-/-}$ (D) hindbrain.

(E–G) Immunostains of transverse sections from WT (E) and *Ntn1*$^{-/-}$ (F) E15.5 embryos. Low- (E and F) and high-power (E'–F'') images of laminin (blue) and DCC (green) show an ectopic break in the BM (yellow arrowheads, F' and F'') in *Ntn1* mutants, quantified in (G) (mean ± SD, Student's t test).

(H–I') Stains on the same WT (H) and mutant (I and I') sections for RC2, a radial glia marker, show that the radial glia endfeet (red) remain attached to the laminin-positive BM (blue) in the mutant, even extending together with PN processes through breaks in the laminin (yellow arrowhead), shown also in a single-channel image for RC2 in (I').

hb, hindbrain; VIII, vestibulocochlear nerve; WT, wild-type.

way into the cochlea in *DCC*$^{-/-}$*; Neo1*$^{Gt/Gt}$ double mutants than in *DCC*$^{-/-}$ single mutants, similar numbers entered the nerve roots in E15.5 animals of both genotypes. Ntn1, therefore, appears to act largely through DCC to prevent PNs from crossing the CNS-PNS boundary but may influence their subsequent behavior through Neogenin expressed in other tissues.

Ntn1$^{-/-}$ Mutants Retain Boundary Cap Cells at Nerve Roots

Our results show that, in addition to its canonical role as a chemoattractant, Ntn1 contributes to CNS-PNS segregation, raising the question of how Ntn1 mediates this function. Many cellular structures contribute to the compartmentalization of the CNS. Neural crest-derived boundary cap cells (BCCs), for example, reside at all spinal nerve roots, and loss of BCCs or the cues they secrete results in the ectopic migration of motor neurons into the ventral root (Bron et al., 2007; Garrett et al., 2016; Mauti et al., 2007; Vermeren et al., 2003). The role of BCCs in the hindbrain is less well understood, though they reside at the trigeminal and facial nerve roots in mice (Garrett et al., 2016) and chicks (Niederländer and Lumsden, 1996). Loss of *Ntn1* could alter the position of BCCs at cranial nerve roots, in turn permitting the departure of CNS neurons along nerves.

To test this possibility, we used RNAscope to detect *Egr2*, one of the only markers for BCCs (Vermeren et al., 2003), and counterstained for laminin to assess the distribution of BCCs at the Vth, VIIth, and VIIIth nerves. At E11.5, when DCC+ processes have already entered the periphery (Figure S1), *Egr2*+ BCCs were observed at nerve entry and exit sites in both controls

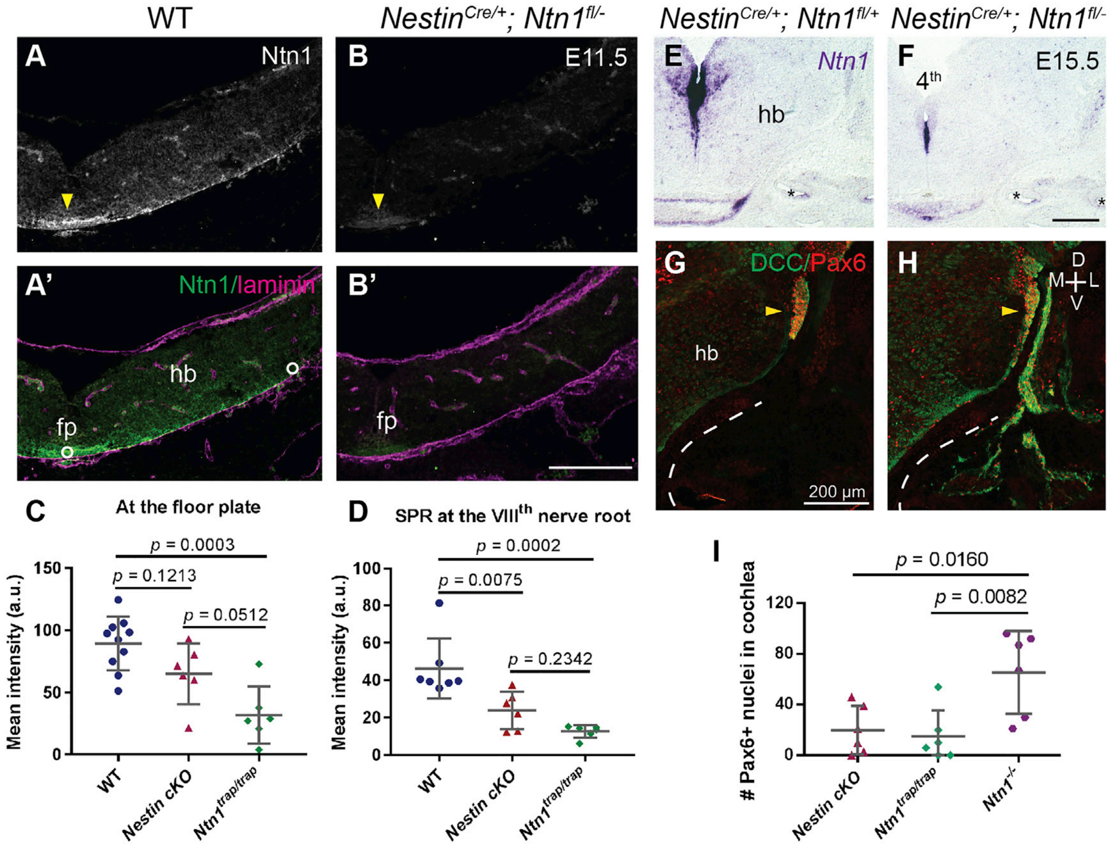

Figure 6. Ntn1 in the SPR, but Not the FP, Is Required for PN Confinement

(A–D) Immunostaining for Ntn1 shows depletion from the SPR of E11.5 *Nestin* cKO animals (B and B') compared to controls (A and A'), with maintained expression in the FP (arrowheads). Ntn1 intensity was measured at the FP or in the SPR (white circles in A'), quantified in (C) and (D), respectively (mean ± SD). For (C), F = 12.06; DF = 19; p = 0.0004; for (D), F = 14.73; DF= 16; p = 0.0002; one-way ANOVA with Tukey's multiple comparisons test.

(E and F) *In situ* hybridization for *Ntn1* further illustrates that relative to *Nestin*$^{Cre/+}$; *Ntn1*$^{fl/+}$ animals (E), *Ntn1* is selectively reduced in the ventricular zone of E15.5 *Nestin* cKO embryos (F).

(G–I) DCC (green) and Pax6 (red) immunostains on E15.5 transverse head sections. *Nestin* cKO animals (H) retain the AES (yellow arrowheads), but it is smaller and deformed compared to controls (G), and there are many Pax6+ nuclei in the cochlea, quantified in (I). Depleting Ntn1 from the SPR is sufficient to partly recapitulate the null phenotype and fully phenocopy the hypomorph (I; mean ± SD) (F = 7.542; DF = 15; p = 0.0054; one-way ANOVA with Tukey's multiple comparisons test). Refer to Figure S4 for raw data for the gene trap allele. Dotted lines indicate the outline of the cochlea.

fp, floor plate; hb, hindbrain; 4th, fourth ventricle. Asterisks indicate cochlear duct. All scale bars indicate 200 µm.

See also Figure S4.

and mutants (Figures 5A–5B''; n = 3 animals per genotype). This result is not unexpected, given that, based on the number of ectopic CNS neurons in the periphery, the *Ntn1*$^{-/-}$ hindbrain phenotype is much more severe than what was previously described in animals lacking BCCs (Vermeren et al., 2003). These data show that Ntn1 maintains the CNS-PNS divide through mechanisms distinct from those of BCCs.

Ectopic Neurons Exit the CNS Independent of Defects in BM Organization

In addition to BCCs, an effective CNS-PNS border depends on BM integrity, which is maintained, in part, by radial glial endfeet lining the pial surface. Deletion of BM components or detachment of radial glial endfeet from the pial surface causes BM rupture, defects in neuronal migration, extrusion of cortical neurons into the subarachnoid space, and ectopic migration of spinal cord motor neurons into the ventral root (Beggs et al., 2003; Halfter et al., 2002; Lee and Song, 2013; Moore et al., 2002; Nakagawa et al., 2015; Satz et al., 2010). Since Netrins affect BM integrity in some tissues (Abraira et al., 2008; Liu et al., 2004; Srinivasan et al., 2003; Yebra et al., 2003; Ziel et al., 2009), we wondered whether loss of *Ntn1* might cause defects in the pial BM or in the organization of the radial glial endfeet, thereby enabling PN exodus.

To assess BM integrity prior to the earliest signs of the phenotype, we performed transmission electron microscopy (TEM) of E10.5 control and *Ntn1*$^{-/-}$ animals. At this age, the BM looks like a thin, diffuse rope surrounding the hindbrain, and we were able to follow the BM from the ventral edge of the VIIIth nerve root to the midline. The appearance of the BM was highly variable, altering in thickness, smoothness, and curvature, with no discernable pattern in WT and null animals (Figures 6C

and 6D; n = 3 WT, 4 *Ntn1*$^{-/-}$). In rare cases, we observed what appeared to be ectopic processes reaching into the periphery, yet the surrounding BM still did not look diminished in a way that would, *a priori*, enable neurons to exit.

Several days later, the BM in *Ntn1*$^{-/-}$ mutants still looked intact overall, as assessed by laminin staining. However, small ectopic breaks were consistently observed near the AES and the vestibulocochlear nerve (Figures 5E–5F''; n = 3 *Ntn1*$^{-/-}$), where ectopic DCC+ processes protrude, resulting in a significant decrease in the area covered by laminin adjacent to the AES (Figure 5G; n = 4 control, 5 *Ntn1*$^{-/-}$ ears). These breaks appeared independent of impaired radial glia architecture, whose RC2/Nestin+ endfeet remained attached to the pial surface in E15.5 mutants, as in controls (Figures 5H and 5I; n = 2 control, 3 *Ntn1*$^{-/-}$). Moreover, at the sites of BM breaks, the radial glial endfeet projected further without showing obvious changes in their morphology or organization (Figure 5I'). Since BM integrity is normal at E10.5, with no apparent changes in radial glia organization at E15.5, it is unlikely that Ntn1 is required for BM integrity *per se*, consistent with the fact that Ntn1 has no effects on laminin assembly *in vitro* (Schneiders et al., 2007). Altogether, the lack of defects in key cell types that contribute to CNS integrity indicate that Ntn1 in the SPR acts directly on migrating neurons to keep them in the CNS.

SPR-Localized *Ntn1* Produced by Hindbrain Progenitors Is Required for Confinement

Our results contrast with those from previous studies that reported no phenotypes in the spiral ganglion of *Ntn1* hypomorphs (Howell et al., 2007; Kim et al., 2016). These differences could be attributed to the hypomorphic nature of the *Ntn1*$^{trap/trap}$ mice, which show a weaker phenotype: many PNs get close to their final destination (Figures S4D and S4D'), and there are significantly fewer Pax6+ cells in E15.5 *Ntn1*$^{trap/trap}$ cochleae (Figures S4B–S4C'; n = 6 cochleae). The number of ectopic neurons remained unchanged at E18.5 (Figure S4E; n = 6 cochleae), further indicating that, unlike null animals, the phenotype does not worsen as later born PNs migrate out. Despite this difference, the pontine nuclei are absent in *Ntn1*$^{trap/trap}$ animals, indicating that confinement and guidance are differentially affected by the loss of Ntn1, possibly due to differences in the availability or localization of Ntn1 *in vivo*.

In the developing hindbrain, Ntn1 is present both in the FP and in the SPR (Figure 1) (Dominici et al., 2017; MacLennan et al., 1997). We wondered whether the role in confinement might be attributed specifically to Ntn1 in the SPR, which is primarily supplied by progenitors in the ventricular zone (Dominici et al., 2017; Varadarajan et al., 2017). Using *Nestin*$^{Cre/+}$ (Zimmerman et al., 1994) and a floxed allele of *Ntn1*, we significantly reduced Ntn1 in the SPR of *Nestin*$^{Cre/+}$; *Ntn1*$^{fl/-}$ (*Nestin* conditional knockout [cKO]) animals (Figures 6A–6D; n = 2 controls and 3 *Nestin* cKO). Despite the presence of residual *Ntn1* protein (Figures 6C and 6D) and transcript (Figures 6E and 6F) at the FP, PNs migrated ectopically into the ear (Figures 6G and 6H; n = 2 control and 3 *Nestin* cKO), partially phenocopying *Ntn1*$^{-/-}$animals and fully phenocopying the hypomorphs (Figure 6I; n = 6 ears per genotype), which showed a similar distribution of Ntn1 protein, i.e., a severe decrease in the SPR (Figure 6D) with residual Ntn1 present at the midline (Figures 6C and S4; n $\geq$ 3 animals per genotype). These results support two conclusions. First, Ntn1 derived from the ventricular zone—which provides most of the Ntn1 in the SPR—ensures the compartmentalization of the CNS and PNS. Second, residual Ntn1 in hypomorphs and from the FP of *Nestin* cKO animals is sufficient to reduce the departure of CNS neurons into the periphery, but not to guide them reliably to the midline, as the pontine nuclei are missing in the hypomorph (Serafini et al., 1996; Yee et al., 1999).

Overexpression of *Ntn1* throughout the CNS Rescues CNS-PNS Boundary Integrity

Our results raise the possibility that Ntn1 serves dual functions in the developing hindbrain, both securing the CNS-PNS boundary and attracting PNs to the ventral midline. In this model, the low levels of Ntn1 that persist in *Ntn1* hypomorphs may be sufficient to establish a partially functional boundary, but not to mediate guidance to the midline, thereby explaining phenotypic differences between the hypomorphic and null mutants. Thus, Ntn1 could act instructively in a gradient to direct PNs to the midline and permissively in the SPR to keep them in the CNS.

To disambiguate these two possible functions, we disrupted Ntn1's role as a guidance cue by altering its pattern of distribution using a Cre-dependent *Ntn1* conditional expressor (*Ntn1*$^{CE/+}$), which produces a myc-tagged chick Ntn1 protein (cNtn1) with the same biological activity as mouse Ntn1 (mNtn1; Serafini et al., 1994). E11.5 *Nestin*$^{Cre/+}$; *Ntn1*$^{CE/+}$ animals showed widespread expression of cNtn1-myc throughout the hindbrain, overlaid on top of endogenous mNtn1 protein in the FP and SPR (Figures 7A–7B'; see also Figure S5; n = 4 *Nestin*$^{Cre/+}$; *Ntn1*$^{CE/+}$). Despite this clear change in Ntn1 protein distribution, PN migration appeared qualitatively normal: PNs reached the midline (Figures 7E and 7F), and no ectopic neurons were observed in the periphery (data not shown). To determine whether the lack of a phenotype reflected a dominant role for endogenous mNtn1, we crossed the *Nestin*$^{Cre/+}$; *Ntn1*$^{CE/+}$ animals onto the *Ntn1*$^{-/-}$ background. Both the *cNtn1* transcript (Figure S6; n = 3 *Nestin*$^{Cre/+}$; *Ntn1*$^{CE/+}$; *Ntn1*$^{-/-}$) and protein (Figures 7C–7D'; n = 2 *Nestin*$^{Cre/+}$; *Ntn1*$^{CE/+}$; *Ntn1*$^{-/-}$) were present throughout the hindbrain, with cNtn1-myc enriched in the SPR but reduced at the FP. Thus, we significantly altered Ntn1 localization, thereby distorting any directional information that might be encoded in a gradient while maintaining a rich source of Ntn1 in the SPR.

Despite the drastic change in Ntn1 distribution, PN migration was surprisingly normal in all *Nestin*$^{Cre/+}$; *Ntn1*$^{CE/+}$; *Ntn1*$^{-/-}$ embryos (n = 4), as evidenced by the presence of both a well-defined AES and PNs at the midline where the pontine nuclei are normally found (Figure 7G). Although some PNs reached the midline in all animals, the extent of rescue varied, even between the two sides of the animal. We observed a complete rescue in 4 out of 8 cases (two per embryo), as defined by a qualitatively normal AES and no PNs detected in the periphery (Figures 7H and 7I). In the other cases, the AES was misshapen and sometimes accompanied by clusters of Pax6+ neurons in the proximal segment of the vestibulocochlear nerve or sparse Pax6+ neurons in other cranial nerves (Figure 7J). Although PNs appear to be resistant to major disruptions in the pattern of *Ntn1* expression, these occasional errors may reflect

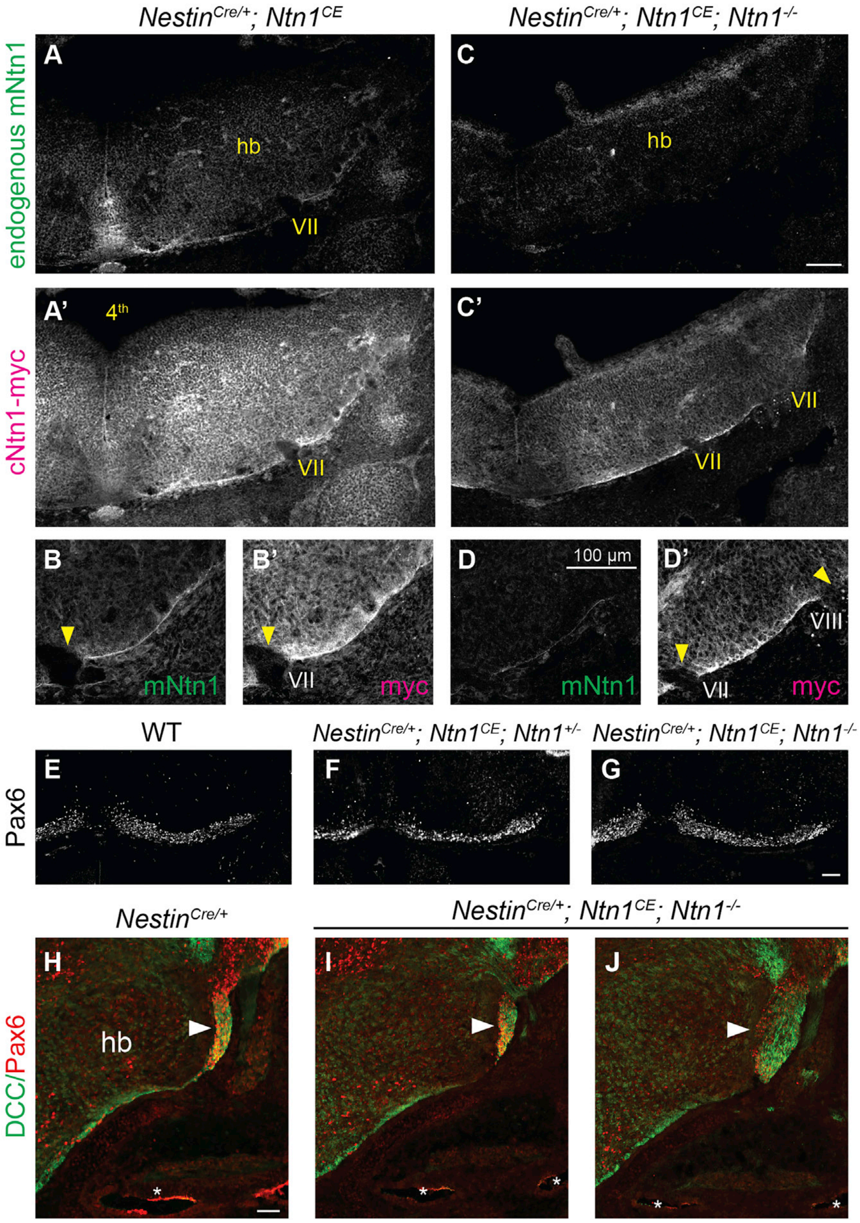

Figure 7. Broadly Expressing *cNtn1* in the Hindbrain Can Rescue Confinement Defects in *Ntn1*$^{-/-}$ Animals

(A–D') Transverse sections through E11.5 conditional expressor tissue immunostained for mNtn1 (A–D) and cNtn1-myc (A'–D'). cNtn1-myc is broadly distributed throughout the hindbrain (A'), overlaid on top of endogenous Ntn1 protein (A). Whereas mNtn1 is enriched at the FP, cNtn1 is relatively reduced, but both are enriched in the SPR (B and B'). Low-power images (C, C') show that a similar distribution of cNtn1 persists in the null background, where despite the absence of mNtn1 (C), cNtn1 is present throughout the hindbrain, with less in the floor plate (C'). High-power images (D, D') show that without mNtn1 (D), cNtn1 is the only Ntn1 enriched in the SPR (D'). In all cases, note the absence of any Ntn1 near nerve entry roots (yellow arrowheads).

(E–J) E15.5 transverse sections immunostained for Pax6 (red) and DCC (green). Single-channel images of Pax6 (E–G) show PNs accumulating at the midline of all conditional expressors, in both *Ntn1*$^{+/-}$ (F) and *Ntn1*$^{-/-}$ (G) backgrounds. Conditional expression of cNtn1-myc rescued confinement in some *Ntn1*$^{-/-}$ animals (I), as shown by a qualitatively normal AES (white arrowheads). In others, we observed a partial rescue in the form of a misshapen AES and a cluster of ectopic neurons in the nerve (J). Control sections at the midline (E) and AES (H) are provided for comparison.

hb, hindbrain; 4th, fourth ventricle. Roman numerals indicate cranial nerves. Asterisks indicate cochlear duct. All scale bars indicate 100 µm.

See also Figures S5 and S6.

some requirement for the WT pattern of *Ntn1* expression. Alternatively, the degree of rescue may be sensitive to slight variations in the timing or efficiency of *Nestin*^{Cre}-mediated recombination. Importantly, none of the embryos contained Pax6+ neurons in the cochlea. Thus, broad expression of Ntn1 is sufficient to restrict PNs from migrating into the periphery, consistent with the model that Ntn1 acts locally to provide a preferred substrate for neuronal migration, thereby keeping neurons confined to the CNS.

DISCUSSION

In the developing hindbrain, rhombic lip-derived neurons migrate long distances to form brainstem nuclei amidst a crowded network of nerves linking the CNS and PNS. We show here that SPR-localized Ntn1 maintains the CNS-PNS divide by preventing these highly motile neurons from straying into cranial nerves and entering the periphery. Our findings point to a model in which Ntn1 in the SPR acts as a preferred substrate for migrating neurons, thereby keeping them away from nerve roots devoid of Ntn1. Like flags marking a hiking trail, Ntn1 facilitates the successful migration of rhombic lip-derived neurons by establishing a preferred corridor for growth. Without this corridor, the neurons wander off trail, losing track of and failing to reach their destination.

In support of the idea that Ntn1 acts in the SPR to keep migrating neurons on track, migrating PNs express the Ntn1 receptor DCC and respond to Ntn1 *in vitro* (Yee et al., 1999). Ntn1 protein is also notably enriched in the SPR but absent at cranial nerve roots, which rhombic lip derivatives avoid. Thus, DCC+ PNs may prefer the Ntn1-rich environment surrounding the nerve roots so much that they reliably migrate around them, with the Ntn1-negative gap discouraging their entry. In agreement with this interpretation, the amount of Ntn1 in the

SPR correlates with the strength of the confinement phenotype. For instance, using *Nestin^{Cre}* to selectively reduce Ntn1 in the SPR but not the FP caused many PNs to enter the periphery. More strikingly, no ectopic PNs were observed in the cochlea when *Nestin^{Cre}* was used to restore cNtn1-myc only to the SPR in the null background, where Ntn1 is never produced by the FP and the broad ectopic distribution of Ntn1 throughout the hindbrain obscures any positional information normally encoded by localized Ntn1. Thus, the pattern of Ntn1 expression does not seem to matter for the confinement of migrating neurons, as long as Ntn1 protein accumulates in the SPR.

Although a direct role for Ntn1 seems most likely, indirect effects might also contribute to the overall phenotype. For example, the departure of PNs could be facilitated by the presence of errant axons from earlier born neurons that breached the CNS-PNS border. However, such a mechanism is unlikely to account for the entire phenotype, as both neuronal cell bodies and processes have already entered the periphery at E11.5 (arrows in Figure S1B''), the earliest point when we can detect a phenotype. Likewise, PNs appear segregated from other DCC+ ectopic axons in the VIIIth nerve, and these ectopias can arise independently (Figure S2). Thus, the departure of neurons does not seem to depend, *a priori*, on the presence of a pre-existing ectopic axon tract. It is, of course, possible that PNs occasionally migrate along earlier born ectopic processes as they escape the CNS, similar to the fasciculation of PN leading processes within the normal AES (Ono and Kawamura, 1990) and of later born axons that follow pioneer axons toward their targets. However, this would not rule out or diminish the role of Ntn1 in the confinement of rhombic lip-derived neurons overall.

In another scenario, SPR-localized Ntn1 could promote or maintain a physically sound CNS-PNS boundary, in addition to affecting neuronal migration. While we cannot rule out subtle changes, the overall organization of the CNS-PNS boundary appeared intact in *Ntn1$^{-/-}$* animals. BCCs were present at nerve roots, and there were no obvious changes in the integrity of the BM surrounding the hindbrain, consistent with the fact that Ntn1 has no effect on BM assembly *in vitro* (Schneiders et al., 2007). These data suggest that Ntn1 acts directly on PNs to corral them within the SPR, thereby preventing them from leaving the CNS altogether. Our findings add to a growing body of evidence supporting a permissive role for Ntn1 (Dominici et al., 2017; Varadarajan et al., 2017; Yamauchi et al., 2017), and they expand the repertoire of Ntn1 functions in the developing nervous system.

Distinct Functions for Ntn1 in Confinement along the Rostrocaudal Axis

Compared to other aspects of neural development, little is known about the initiation or maintenance of the CNS-PNS boundary, which selectively permits the passage of neural processes—but not cell bodies—into peripheral nerves. Studies in the spinal cord have highlighted the importance of BCCs (Vermeren et al., 2003) and chemorepellents such as Ntn5 (Garrett et al., 2016), Sema3B, Sema3G, and Sema6A (Bron et al., 2007; Mauti et al., 2007) in retaining motor neuron cell bodies inside the CNS, even as they extend their axons out to the periphery. In stark contrast, confinement phenotypes have not been reported in the hindbrain, although BCCs express similar repellents at cranial nerve roots. For example, we found no evidence of ectopic Pax6+ neurons in the cochlea of *Ntn5$^{-/-}$* mice (data not shown), despite the presence of ectopic motor neurons in the spinal cord (Garrett et al., 2016). This discrepancy underscores two points. First, the molecular mechanisms that define the CNS-PNS boundary in the vertebrate brainstem remain unknown; and second, the hindbrain and the spinal cord may have evolved unique ways of maintaining the CNS-PNS boundary.

Indeed, our work illustrates that the same molecule may have distinct functions in hindbrain versus spinal cord confinement. In the spinal cord, Ntn1 plays a relatively limited role, preventing the axons of a single population of neurons from straying into the periphery. Notably, the cell bodies do not follow in *Ntn1* mutants (Laumonnerie et al., 2014). Thus, in this context, the misrouting of CNS axons into the PNS is much like other axon guidance phenotypes. In contrast, in the hindbrain, Ntn1 signaling appears to play an integral role in defining the CNS-PNS boundary, as evidenced by both the sheer number of neurons exiting the CNS and, most importantly, the departure of cell bodies, which an effective CNS-PNS boundary absolutely forbids. These differences in Ntn1 function may reflect the distinct developmental demands of the two brain regions: whereas migration is limited in the spinal cord, there is extensive migration of multiple populations of neurons over long distances and past multiple nerve roots in the hindbrain. As such, having a centrally derived cue play a weightier role in confinement may offer the greater fidelity and robustness needed for rhombic-lip derivatives to complete their migratory routes successfully. It remains to be seen whether another centrally derived cue might play a more prominent role in confinement in the spinal cord, particularly since most motor neurons stay within the CNS, even when all BCCs are ablated (Vermeren et al., 2003).

Cell-Autonomous and Non-Cell-Autonomous Functions for Multiple Ntn1 Receptors in Confinement

Our findings provide additional evidence for Ntn1's multifunctionality, which likely depends on its diverse repertoire of receptors. PNs express receptors mediating both attraction, such as DCC, and repulsion, such as Unc5B/C. However, Ntn1-mediated confinement does not seem to depend on repulsion, since PNs remain within the CNS in *Unc5b* and *Unc5c* mutants (Di Meglio et al., 2013; Kim and Ackerman, 2011). Moreover, both HoxB4+ (Unc5B-low) and HoxB4− (Unc5B-high) PNs (Di Meglio et al., 2013) escape into the periphery in *Ntn1* hypomorphs (Figure S7), indicating that differential responsiveness to Ntn1 cannot account for the partial phenotype. Thus, Unc5B/Cs appear to influence only the later stages of Ntn1-mediated PN migration, comparable to the way Unc5A/Cs position commissural neuron cell bodies in the spinal cord but are not required for confinement of their axons (Laumonnerie et al., 2014).

In contrast, as an obligate receptor expressed on commissural axons and PNs, DCC mediates confinement in both the spinal cord and hindbrain. We also discovered a surprising role for Neogenin in *trans*, as *Neo1* is neither expressed nor required in migrating PNs, though it is present at low levels throughout the neuroepithelium and at higher levels on cranial nerves and in

the surrounding mesenchyme. These data suggest that complete containment depends both on Ntn1-DCC signaling within PNs and also on Ntn1-receptor interactions in the environment. Since Ntn1-Neogenin interactions mediate adhesion in the developing mammary gland (Srinivasan et al., 2003), similar interactions in the BM around the VIIIth nerve could prevent movement into the nerve root, providing an additional safeguard for CNS-PNS segregation. However, any effects of Ntn1 signaling on the structural integrity of the CNS-PNS border are likely to be subtle, as the BM did not appear strikingly different in *Ntn1*$^{-/-}$ mutants by electron microscopy (EM) or immunostaining. Moreover, PNs stay confined to the CNS in *ISPD* mutants (data not shown), which have fragmented BMs (Wright et al., 2012). Hence, disrupting boundaries alone is not sufficient to induce the departure of CNS neurons, indicating that Ntn1 plays an active signaling role across multiple cell types in confining migrating neurons to the CNS.

Finding Unity in Ntn1's Diverse Functions: a Role for Locally Produced Ntn1 in Neural Development

In addition to being the archetype of diffusible guidance cues, much of Ntn1's prominence can be attributed to its versatility. Beyond its role in axon guidance, cell migration, and confinement, Ntn1 modulates angiogenesis and tissue morphogenesis, cell adhesion, synapse formation, and cell survival in cancer (reviewed in Cirulli and Yebra, 2007; Lai Wing Sun et al., 2011). Historically, a large emphasis has been placed on the division between long- and short-range functions, which are categorized based on where Ntn1 acts relative to the source of its expression. For instance, textbook models of Ntn1 as a long-range attractant depict commissural axons navigating along an increasing gradient of FP-derived Ntn1 in the spinal cord. The situation *in vivo*, however, is more complicated. Although Ntn1 is distributed in a gradient in the spinal cord (Kennedy et al., 2006) and can act over a distance *in vitro* (Kennedy et al., 1994; Yee et al., 1999), it was purified as a heparin-binding protein (Serafini et al., 1994) and found to interact with BM components, including type IV collagen and heparin sulfate proteoglycans (Geisbrecht et al., 2003; Geisen et al., 2008; Kappler et al., 2000). This had raised the possibility that it might function at both short- and long-range (Serafini et al., 1994; Kennedy et al., 1994), and a local role in short-range guidance was soon demonstrated at the optic nerve head (Deiner et al., 1997). Short-range functions have also been demonstrated during tissue morphogenesis, such as BM breakdown in the inner ear (Nishitani et al., 2017; Salminen et al., 2000) or adhesion between two cell layers in the mammary gland (Srinivasan et al., 2003).

Our findings add to a growing body of work that suggest that many of Ntn1's other functions in the nervous system may be grounded in local signaling, a shared mechanism that may provide a foundation for its diverse roles. Membrane-tethered versions of Ntn, for example, can rescue guidance defects in the *Drosophila* nerve cord and visual system that were previously ascribed to soluble Ntn (Brankatschk and Dickson, 2006; Timofeev et al., 2012). More recently, several groups have demonstrated that commissural guidance depends on ventricular-zone-derived Ntn1 accumulating in the SPR and along the commissural axons (Dominici et al., 2017; Varadarajan et al., 2017; Yamauchi et al., 2017), expanding on related observations (Charron et al., 2003; Kennedy et al., 2006). We have similarly revealed a role for SPR-localized Ntn1 in cellular confinement, providing an alternative explanation for both the reduced number of PNs in *Ntn1*$^{trap/trap}$ mice, which was thought to reflect Ntn1's tropic and trophic roles (Yee et al., 1999), and the presence of ectopic neurons in *DCC*$^{-/-}$ cochleae, which was attributed to a mis-positioning of SGNs (Kim et al., 2016). Thus, across multiple species and in multiple regions of the nervous system, Ntn1 appears to act locally to mediate its purported long-range functions.

Given the clear importance of Ntn1 for nervous system wiring, the possibility that Ntn1 may not act as a long-range instructive cue for PNs raises the question of where the directional information comes from. One idea is that a gradient of Ntn1 activity is achieved through interactions with other cues in the environment. Indeed, every confirmed Ntn1 receptor also interacts with additional ligands (Ahmed et al., 2011; Karaulanov et al., 2009; Rajagopalan et al., 2004; Yamagishi et al., 2011), raising the possibility that Ntn1 is a crucial collaborator for many guidance pathways, perhaps mediating short-range interactions that are necessary for axons to grow reliably toward other ligands. This could occur either directly, i.e., by binding to the same receptors, or indirectly, i.e., by attaching migrating neurons to the BM, where they may be steered by other cues such as Slits. This may explain Ntn1's ability to augment the effect of other guidance molecules synergistically (reviewed in Morales and Kania, 2017). Thus, even 20 years after its discovery, Ntn1 continues to inform new models for how the complex networks of the nervous system are constructed reliably and accurately using relatively few guidance cues.

EXPERIMENTAL PROCEDURES

Further details and an outline of resources used in this work can be found in the Supplemental Experimental Procedures.

Animal Models

The following mouse strains were used and genotyped as described previously: *Ntn1*$^{fl/fl}$, *Ntn1*$^{+/-}$ (Yung et al., 2015), *Ntn1*$^{CE/+}$ (Nishitani et al., 2017), *DCC*$^{+/-}$; *Neo1*$^{Gt/+}$ (Fazeli et al., 1997; Leighton et al., 2001; Xu et al., 2014), *Neo1*$^{-/-}$ and *Neo1*$^{fl/fl}$ (Kam et al., 2016), *Atoh1*^{CreERT2} (Machold and Fishell, 2005), *Six3*^{Cre} (Furuta et al., 2000), *Nestin*^{Cre} (Tronche et al., 1999), *MafB*^{GFP} (Moriguchi et al., 2006), and *Ai14* Cre-dependent *tdTomato* (Madisen et al., 2010).

Mice were maintained on a C57BL/6 background. Noon on the day of the plug was considered E0.5. Tamoxifen (Sigma-Aldrich) injections were carried out at 20 mg/mL in sunflower oil at 1 mg/10 g of body weight. Since all experiments were performed on embryonic mice, whole litters, which included both male and female mice, were used for experiments. We did not detect any sex-based differences in our phenotype. The ages used in each experiment are included in the relevant text, figures, and figure legends. Experiments were performed with the observer blind to genotype, though the ensuing image analyses were not due to the obvious nature of the phenotypes. All animal experiments were approved by the Institutional Animal Use Care Committee at Harvard Medical School.

Statistical Analysis

All statistical comparisons were done using Prism software (GraphPad, La Jolla, CA, USA) and presented as mean ± SD. Statistical significance was determined by a Student's t test when comparing between two groups. If more than two groups were being considered, a one-way ANOVA was

performed with Tukey's multiple comparisons test. In cases of the latter, the multiplicity adjusted p values were included in the figures, and the p value of the ANOVA was reported in the figure legends. Sample size for all experiments was determined empirically based on standards in the field. Specific details for each experiment are included in the text (n values and their meanings) or in the figure legends (statistical tests used).

SUPPLEMENTAL INFORMATION

Supplemental Information includes Supplemental Experimental Procedures and seven figures and can be found with this article online at https://doi.org/10.1016/j.celrep.2018.01.068.

ACKNOWLEDGMENTS

We thank the Neurobiology Department; the Neurobiology Imaging Facility (NINDS P30 NS072030); the HMS Electron Microscopy Core Facility; Drs. Kevin Wright (OHSU) and Rob Burgess (The Jackson Laboratory) for embryonic tissue; Jocelyn Curran and Emilie Dumontier for genotyping assistance; and Dr. Elio Raviola for help with EM analysis. We are also grateful to Dr. Alain Chédotal for sharing unpublished results. This work was supported by NIH grant R21 DC014916 (to L.V.G.), by NIDCD training grants F31 DC014603 and T32 DC000038 (to A.R.Y.), and by CIHR grants MOP136872 and MOP130387 (to J.-F.C.). Z.W. and M.T.L. were supported by the Rockefeller University and Stanford University.

AUTHOR CONTRIBUTIONS

A.R.Y. and L.V.G. designed the research, analyzed the results, and wrote the manuscript. N.R.D. discovered the phenotype and A.R.Y. performed all the experiments. J.-F.C. provided the *Wnt1*^{Cre}*; DCC*$^{-/-}$*; Neo1*$^{fl/fl}$, and *Neo1*$^{-/-}$ tissue. Z.W. and M.T.-L. provided the *DCC*$^{+/-}$*; Neo1*$^{Gt/+}$ mice and tissue. J.-F.C., Z.W., and M.T.-L. also shaped the course of the study and provided input on the manuscript.

DECLARATION OF INTERESTS

The authors declare no competing interests.

Received: June 30, 2017
Revised: December 13, 2017
Accepted: January 22, 2018
Published: February 13, 2018

REFERENCES

Abraira, V.E., Del Rio, T., Tucker, A.F., Slonimsky, J., Keirnes, H.L., and Goodrich, L.V. (2008). Cross-repressive interactions between Lrig3 and netrin 1 shape the architecture of the inner ear. Development *135*, 4091–4099.

Ahmed, G., Shinmyo, Y., Ohta, K., Islam, S.M., Hossain, M., Naser, I.B., Riyadh, M.A., Su, Y., Zhang, S., Tessier-Lavigne, M., and Tanaka, H. (2011). Draxin inhibits axonal outgrowth through the netrin receptor DCC. J. Neurosci. *31*, 14018–14023.

Alcántara, S., Ruiz, M., De Castro, F., Soriano, E., and Sotelo, C. (2000). Netrin 1 acts as an attractive or as a repulsive cue for distinct migrating neurons during the development of the cerebellar system. Development *127*, 1359–1372.

Bae, G.-U., Yang, Y.-J., Jiang, G., Hong, M., Lee, H.-J., Tessier-Lavigne, M., Kang, J.-S., and Krauss, R.S. (2009). Neogenin regulates skeletal myofiber size and focal adhesion kinase and extracellular signal-regulated kinase activities in vivo and in vitro. Mol. Biol. Cell *20*, 4920–4931.

Beggs, H.E., Schahin-Reed, D., Zang, K., Goebbels, S., Nave, K.A., Gorski, J., Jones, K.R., Sretavan, D., and Reichardt, L.F. (2003). FAK deficiency in cells contributing to the basal lamina results in cortical abnormalities resembling congenital muscular dystrophies. Neuron *40*, 501–514.

Bin, J.M., Han, D., Lai Wing Sun, K., Croteau, L.-P., Dumontier, E., Cloutier, J.-F., Kania, A., and Kennedy, T.E. (2015). Complete loss of netrin-1 results in embryonic lethality and severe axon guidance defects without increased neural cell death. Cell Rep. *12*, 1099–1106.

Bloch-Gallego, E., Ezan, F., Tessier-Lavigne, M., and Sotelo, C. (1999). Floor plate and netrin-1 are involved in the migration and survival of inferior olivary neurons. J. Neurosci. *19*, 4407–4420.

Brankatschk, M., and Dickson, B.J. (2006). Netrins guide *Drosophila* commissural axons at short range. Nat. Neurosci. *9*, 188–194.

Bron, R., Vermeren, M., Kokot, N., Andrews, W., Little, G.E., Mitchell, K.J., and Cohen, J. (2007). Boundary cap cells constrain spinal motor neuron somal migration at motor exit points by a semaphorin-plexin mechanism. Neural Dev. *2*, 21.

Brugeaud, A., Tong, M., Luo, L., and Edge, A.S.B. (2014). Inhibition of repulsive guidance molecule, RGMa, increases afferent synapse formation with auditory hair cells. Dev. Neurobiol. *74*, 457–466.

Causeret, F., Danne, F., Ezan, F., Sotelo, C., and Bloch-Gallego, E. (2002). Slit antagonizes netrin-1 attractive effects during the migration of inferior olivary neurons. Dev. Biol. *246*, 429–440.

Charron, F., Stein, E., Jeong, J., McMahon, A.P., and Tessier-Lavigne, M. (2003). The morphogen sonic hedgehog is an axonal chemoattractant that collaborates with netrin-1 in midline axon guidance. Cell *113*, 11–23.

Cirulli, V., and Yebra, M. (2007). Netrins: beyond the brain. Nat. Rev. Mol. Cell Biol. *8*, 296–306.

de Diego, I., Kyriakopoulou, K., Karagogeos, D., and Wassef, M. (2002). Multiple influences on the migration of precerebellar neurons in the caudal medulla. Development *129*, 297–306.

Deiner, M.S., Kennedy, T.E., Fazeli, A., Serafini, T., Tessier-Lavigne, M., and Sretavan, D.W. (1997). Netrin-1 and DCC mediate axon guidance locally at the optic disc: loss of function leads to optic nerve hypoplasia. Neuron *19*, 575–589.

Di Meglio, T., Kratochwil, C.F., Vilain, N., Loche, A., Vitobello, A., Yonehara, K., Hrycaj, S.M., Roska, B., Peters, A.H.F.M., Eichmann, A., et al. (2013). Ezh2 orchestrates topographic migration and connectivity of mouse precerebellar neurons. Science *339*, 204–207.

Dominici, C., Moreno-Bravo, J.A., Puiggros, S.R., Rappeneau, Q., Rama, N., Vieugue, P., Bernet, A., Mehlen, P., and Chédotal, A. (2017). Floor-plate-derived netrin-1 is dispensable for commissural axon guidance. Nature *545*, 350–354.

Farago, A.F., Awatramani, R.B., and Dymecki, S.M. (2006). Assembly of the brainstem cochlear nuclear complex is revealed by intersectional and subtractive genetic fate maps. Neuron *50*, 205–218.

Fazeli, A., Dickinson, S.L., Hermiston, M.L., Tighe, R.V., Steen, R.G., Small, C.G., Stoeckli, E.T., Keino-Masu, K., Masu, M., Rayburn, H., et al. (1997). Phenotype of mice lacking functional Deleted in colorectal cancer (Dcc) gene. Nature *386*, 796–804.

Fitzgerald, D.P., Bradford, D., and Cooper, H.M. (2007). Neogenin is expressed on neurogenic and gliogenic progenitors in the embryonic and adult central nervous system. Gene Expr. Patterns *7*, 784–792.

Furuta, Y., Lagutin, O., Hogan, B.L.M., and Oliver, G.C. (2000). Retina- and ventral forebrain-specific Cre recombinase activity in transgenic mice. Genesis *26*, 130–132.

Garrett, A.M., Jucius, T.J., Sigaud, L.P.R., Tang, F.-L., Xiong, W.-C., Ackerman, S.L., and Burgess, R.W. (2016). Analysis of expression pattern and genetic deletion of Netrin5 in the developing mouse. Front. Mol. Neurosci. *9*, 3.

Geisbrecht, B.V., Dowd, K.A., Barfield, R.W., Longo, P.A., and Leahy, D.J. (2003). Netrin binds discrete subdomains of DCC and UNC5 and mediates interactions between DCC and heparin. J. Biol. Chem. *278*, 32561–32568.

Geisen, M.J., Di Meglio, T., Pasqualetti, M., Ducret, S., Brunet, J.-F., Chedotal, A., and Rijli, F.M. (2008). Hox paralog group 2 genes control the migration of mouse pontine neurons through slit-robo signaling. PLoS Biol. *6*, e142.

Halfter, W., Dong, S., Yip, Y.-P., Willem, M., and Mayer, U. (2002). A critical function of the pial basement membrane in cortical histogenesis. J. Neurosci. *22*, 6029–6040.

Howell, D.M., Morgan, W.J., Jarjour, A.A., Spirou, G.A., Berrebi, A.S., Kennedy, T.E., and Mathers, P.H. (2007). Molecular guidance cues necessary for axon pathfinding from the ventral cochlear nucleus. J. Comp. Neurol. *504*, 533–549.

Kam, J.W.K., Dumontier, E., Baim, C., Brignall, A.C., Mendes da Silva, D., Cowan, M., Kennedy, T.E., and Cloutier, J.-F. (2016). RGMB and neogenin control cell differentiation in the developing olfactory epithelium. Development *143*, 1534–1546.

Kappler, J., Franken, S., Junghans, U., Hoffmann, R., Linke, T., Müller, H.W., and Koch, K.W. (2000). Glycosaminoglycan-binding properties and secondary structure of the C-terminus of netrin-1. Biochem. Biophys. Res. Commun. *271*, 287–291.

Karaulanov, E., Böttcher, R.T., Stannek, P., Wu, W., Rau, M., Ogata, S., Cho, K.W.Y., and Niehrs, C. (2009). Unc5B interacts with FLRT3 and Rnd1 to modulate cell adhesion in Xenopus embryos. PLoS ONE *4*, e5742.

Kennedy, T.E., Serafini, T., de la Torre, J.R., and Tessier-Lavigne, M. (1994). Netrins are diffusible chemotropic factors for commissural axons in the embryonic spinal cord. Cell *78*, 425–435.

Kennedy, T.E., Wang, H., Marshall, W., and Tessier-Lavigne, M. (2006). Axon guidance by diffusible chemoattractants: a gradient of netrin protein in the developing spinal cord. J. Neurosci. *26*, 8866–8874.

Kim, D., and Ackerman, S.L. (2011). The UNC5C netrin receptor regulates dorsal guidance of mouse hindbrain axons. J. Neurosci. *31*, 2167–2179.

Kim, Y.J., Wang, S.-Z., Tymanskyj, S., Ma, L., Tao, H.W., and Zhang, L.I. (2016). Dcc mediates functional assembly of peripheral auditory circuits. Sci. Rep. *6*, 23799.

Kratochwil, C.F., Maheshwari, U., and Rijli, F.M. (2017). The long journey of pontine nuclei neurons: from rhombic lip to cortico-ponto-cerebellar circuitry. Front. Neural Circuits *11*, 33.

Lai Wing Sun, K., Correia, J.P., and Kennedy, T.E. (2011). Netrins: versatile extracellular cues with diverse functions. Development *138*, 2153–2169.

Laumonnerie, C., Da Silva, R.V., Kania, A., and Wilson, S.I. (2014). Netrin 1 and Dcc signalling are required for confinement of central axons within the central nervous system. Development *141*, 594–603.

Lee, H., and Song, M.-R. (2013). The structural role of radial glial endfeet in confining spinal motor neuron somata is controlled by the Reelin and Notch pathways. Exp. Neurol. *249*, 83–94.

Leighton, P.A., Mitchell, K.J., Goodrich, L.V., Lu, X., Pinson, K., Scherz, P., Skarnes, W.C., and Tessier-Lavigne, M. (2001). Defining brain wiring patterns and mechanisms through gene trapping in mice. Nature *410*, 174–179.

Liu, Y., Stein, E., Oliver, T., Li, Y., Brunken, W.J., Koch, M., Tessier-Lavigne, M., and Hogan, B.L.M. (2004). Novel role for Netrins in regulating epithelial behavior during lung branching morphogenesis. Curr. Biol. *14*, 897–905.

Machold, R., and Fishell, G. (2005). Math1 is expressed in temporally discrete pools of cerebellar rhombic-lip neural progenitors. Neuron *48*, 17–24.

MacLennan, A.J., McLaurin, D.L., Marks, L., Vinson, E.N., Pfeifer, M., Szulc, S.V., Heaton, M.B., and Lee, N. (1997). Immunohistochemical localization of netrin-1 in the embryonic chick nervous system. J. Neurosci. *17*, 5466–5479.

Madisen, L., Zwingman, T.A., Sunkin, S.M., Oh, S.W., Zariwala, H.A., Gu, H., Ng, L.L., Palmiter, R.D., Hawrylycz, M.J., Jones, A.R., et al. (2010). A robust and high-throughput Cre reporting and characterization system for the whole mouse brain. Nat. Neurosci. *13*, 133–140.

Marcos, S., Backer, S., Causeret, F., Tessier-Lavigne, M., and Bloch-Gallego, E. (2009). Differential roles of Netrin-1 and its receptor DCC in inferior olivary neuron migration. Mol. Cell. Neurosci. *41*, 429–439.

Mauti, O., Domanitskaya, E., Andermatt, I., Sadhu, R., and Stoeckli, E.T. (2007). Semaphorin6A acts as a gate keeper between the central and the peripheral nervous system. Neural Dev. *2*, 28.

Moore, S.A., Saito, F., Chen, J., Michele, D.E., Henry, M.D., Messing, A., Cohn, R.D., Ross-Barta, S.E., Westra, S., Williamson, R.A., et al. (2002). Deletion of brain dystroglycan recapitulates aspects of congenital muscular dystrophy. Nature *418*, 422–425.

Morales, D., and Kania, A. (2017). Cooperation and crosstalk in axon guidance cue integration: additivity, synergy, and fine-tuning in combinatorial signaling. Dev. Neurobiol. *77*, 891–904.

Moriguchi, T., Hamada, M., Morito, N., Terunuma, T., Hasegawa, K., Zhang, C., Yokomizo, T., Esaki, R., Kuroda, E., Yoh, K., et al. (2006). MafB is essential for renal development and F4/80 expression in macrophages. Mol. Cell. Biol. *26*, 5715–5727.

Nakagawa, N., Yagi, H., Kato, K., Takematsu, H., and Oka, S. (2015). Ectopic clustering of Cajal-Retzius and subplate cells is an initial pathological feature in Pomgnt2-knockout mice, a model of dystroglycanopathy. Sci. Rep. *5*, 11163.

Nichols, D.H., and Bruce, L.L. (2006). Migratory routes and fates of cells transcribing the Wnt-1 gene in the murine hindbrain. Dev. Dyn. *235*, 285–300.

Niederländer, C., and Lumsden, A. (1996). Late emigrating neural crest cells migrate specifically to the exit points of cranial branchiomotor nerves. Development *122*, 2367–2374.

Nishitani, A.M., Ohta, S., Yung, A.R., Del Rio, T., Gordon, M.I., Abraira, V.E., Avilés, E.C., Schoenwolf, G.C., Fekete, D.M., and Goodrich, L.V. (2017). Distinct functions for netrin 1 in chicken and murine semicircular canal morphogenesis. Development *144*, 3349–3360.

Ono, K., and Kawamura, K. (1990). Mode of neuronal migration of the pontine stream in fetal mice. Anat. Embryol. (Berl.) *182*, 11–19.

Rajagopalan, S., Deitinghoff, L., Davis, D., Conrad, S., Skutella, T., Chedotal, A., Mueller, B.K., and Strittmatter, S.M. (2004). Neogenin mediates the action of repulsive guidance molecule. Nat. Cell Biol. *6*, 756–762.

Ray, R.S., and Dymecki, S.M. (2009). Rautenlippe redux – toward a unified view of the precerebellar rhombic lip. Curr. Opin. Cell Biol. *21*, 741–747.

Riyadh, M.A., Shinmyo, Y., Ohta, K., and Tanaka, H. (2014). Inhibitory effects of draxin on axonal outgrowth and migration of precerebellar neurons. Biochem. Biophys. Res. Commun. *449*, 169–174.

Salminen, M., Meyer, B.I., Bober, E., and Gruss, P. (2000). Netrin 1 is required for semicircular canal formation in the mouse inner ear. Development *127*, 13–22.

Satz, J.S., Ostendorf, A.P., Hou, S., Turner, A., Kusano, H., Lee, J.C., Turk, R., Nguyen, H., Ross-Barta, S.E., Westra, S., et al. (2010). Distinct functions of glial and neuronal dystroglycan in the developing and adult mouse brain. J. Neurosci. *30*, 14560–14572.

Schneiders, F.I., Maertens, B., Böse, K., Li, Y., Brunken, W.J., Paulsson, M., Smyth, N., and Koch, M. (2007). Binding of netrin-4 to laminin short arms regulates basement membrane assembly. J. Biol. Chem. *282*, 23750–23758.

Serafini, T., Kennedy, T.E., Galko, M.J., Mirzayan, C., Jessell, T.M., and Tessier-Lavigne, M. (1994). The netrins define a family of axon outgrowth-promoting proteins homologous to C. elegans UNC-6. Cell *78*, 409–424.

Serafini, T., Colamarino, S.A., Leonardo, E.D., Wang, H., Beddington, R., Skarnes, W.C., and Tessier-Lavigne, M. (1996). Netrin-1 is required for commissural axon guidance in the developing vertebrate nervous system. Cell *87*, 1001–1014.

Srinivasan, K., Strickland, P., Valdes, A., Shin, G.C., and Hinck, L. (2003). Netrin-1/neogenin interaction stabilizes multipotent progenitor cap cells during mammary gland morphogenesis. Dev. Cell *4*, 371–382.

Timofeev, K., Joly, W., Hadjieconomou, D., and Salecker, I. (2012). Localized netrins act as positional cues to control layer-specific targeting of photoreceptor axons in *Drosophila*. Neuron *75*, 80–93.

Tronche, F., Kellendonk, C., Kretz, O., Gass, P., Anlag, K., Orban, P.C., Bock, R., Klein, R., and Schütz, G. (1999). Disruption of the glucocorticoid receptor gene in the nervous system results in reduced anxiety. Nat. Genet. *23*, 99–103.

van den Heuvel, D.M.A., Hellemons, A.J.C.G.M., and Pasterkamp, R.J. (2013). Spatiotemporal expression of repulsive guidance molecules (RGMs) and their receptor neogenin in the mouse brain. PLoS ONE *8*, e55828.

Varadarajan, S.G., Kong, J.H., Phan, K.D., Kao, T.-J., Panaitof, S.C., Cardin, J., Eltzschig, H., Kania, A., Novitch, B.G., and Butler, S.J. (2017). Netrin1 produced by neural progenitors, not floor plate cells, is required for axon guidance in the spinal cord. Neuron *94*, 790–799.e3.

Vermeren, M., Maro, G.S., Bron, R., McGonnell, I.M., Charnay, P., Topilko, P., and Cohen, J. (2003). Integrity of developing spinal motor columns is regulated by neural crest derivatives at motor exit points. Neuron *37*, 403–415.

Wang, V.Y., Rose, M.F., and Zoghbi, H.Y. (2005). Math1 expression redefines the rhombic lip derivatives and reveals novel lineages within the brainstem and cerebellum. Neuron *48*, 31–43.

Wright, K.M., Lyon, K.A., Leung, H., Leahy, D.J., Ma, L., and Ginty, D.D. (2012). Dystroglycan organizes axon guidance cue localization and axonal pathfinding. Neuron *76*, 931–944.

Xu, K., Wu, Z., Renier, N., Antipenko, A., Tzvetkova-Robev, D., Xu, Y., Minchenko, M., Nardi-Dei, V., Rajashankar, K.R., Himanen, J., et al. (2014). Neural migration. Structures of netrin-1 bound to two receptors provide insight into its axon guidance mechanism. Science *344*, 1275–1279.

Yamagishi, S., Hampel, F., Hata, K., Del Toro, D., Schwark, M., Kvachnina, E., Bastmeyer, M., Yamashita, T., Tarabykin, V., Klein, R., and Egea, J. (2011). FLRT2 and FLRT3 act as repulsive guidance cues for Unc5-positive neurons. EMBO J. *30*, 2920–2933.

Yamauchi, K., Yamazaki, M., Abe, M., Sakimura, K., Lickert, H., Kawasaki, T., Murakami, F., and Hirata, T. (2017). Netrin-1 derived from the ventricular zone, but not the floor plate, directs hindbrain commissural axons to the ventral midline. Sci. Rep. *7*, 11992.

Yebra, M., Montgomery, A.M.P., Diaferia, G.R., Kaido, T., Silletti, S., Perez, B., Just, M.L., Hildbrand, S., Hurford, R., Florkiewicz, E., et al. (2003). Recognition of the neural chemoattractant Netrin-1 by integrins alpha6beta4 and alpha3beta1 regulates epithelial cell adhesion and migration. Dev. Cell *5*, 695–707.

Yee, K.T., Simon, H.H., Tessier-Lavigne, M., and O'Leary, D.M. (1999). Extension of long leading processes and neuronal migration in the mammalian brain directed by the chemoattractant netrin-1. Neuron *24*, 607–622.

Yung, A.R., Nishitani, A.M., and Goodrich, L.V. (2015). Phenotypic analysis of mice completely lacking netrin 1. Development *142*, 3686–3691.

Zelina, P., Blockus, H., Zagar, Y., Péres, A., Friocourt, F., Wu, Z., Rama, N., Fouquet, C., Hohenester, E., Tessier-Lavigne, M., et al. (2014). Signaling switch of the axon guidance receptor Robo3 during vertebrate evolution. Neuron *84*, 1258–1272.

Zhu, Y., Matsumoto, T., Mikami, S., Nagasawa, T., and Murakami, F. (2009). SDF1/CXCR4 signalling regulates two distinct processes of precerebellar neuronal migration and its depletion leads to abnormal pontine nuclei formation. Development *136*, 1919–1928.

Ziel, J.W., Hagedorn, E.J., Audhya, A., and Sherwood, D.R. (2009). UNC-6 (netrin) orients the invasive membrane of the anchor cell in *C. elegans*. Nat. Cell Biol. *11*, 183–189.

Zimmerman, L., Parr, B., Lendahl, U., Cunningham, M., McKay, R., Gavin, B., Mann, J., Vassileva, G., and McMahon, A. (1994). Independent regulatory elements in the nestin gene direct transgene expression to neural stem cells or muscle precursors. Neuron *12*, 11–24.

Cell Reports, Volume *22*

Supplemental Information

Netrin-1 Confines Rhombic

Lip-Derived Neurons to the CNS

Andrea R. Yung, Noah R. Druckenbrod, Jean-François Cloutier, Zhuhao Wu, Marc Tessier-Lavigne, and Lisa V. Goodrich

Supplemental Information

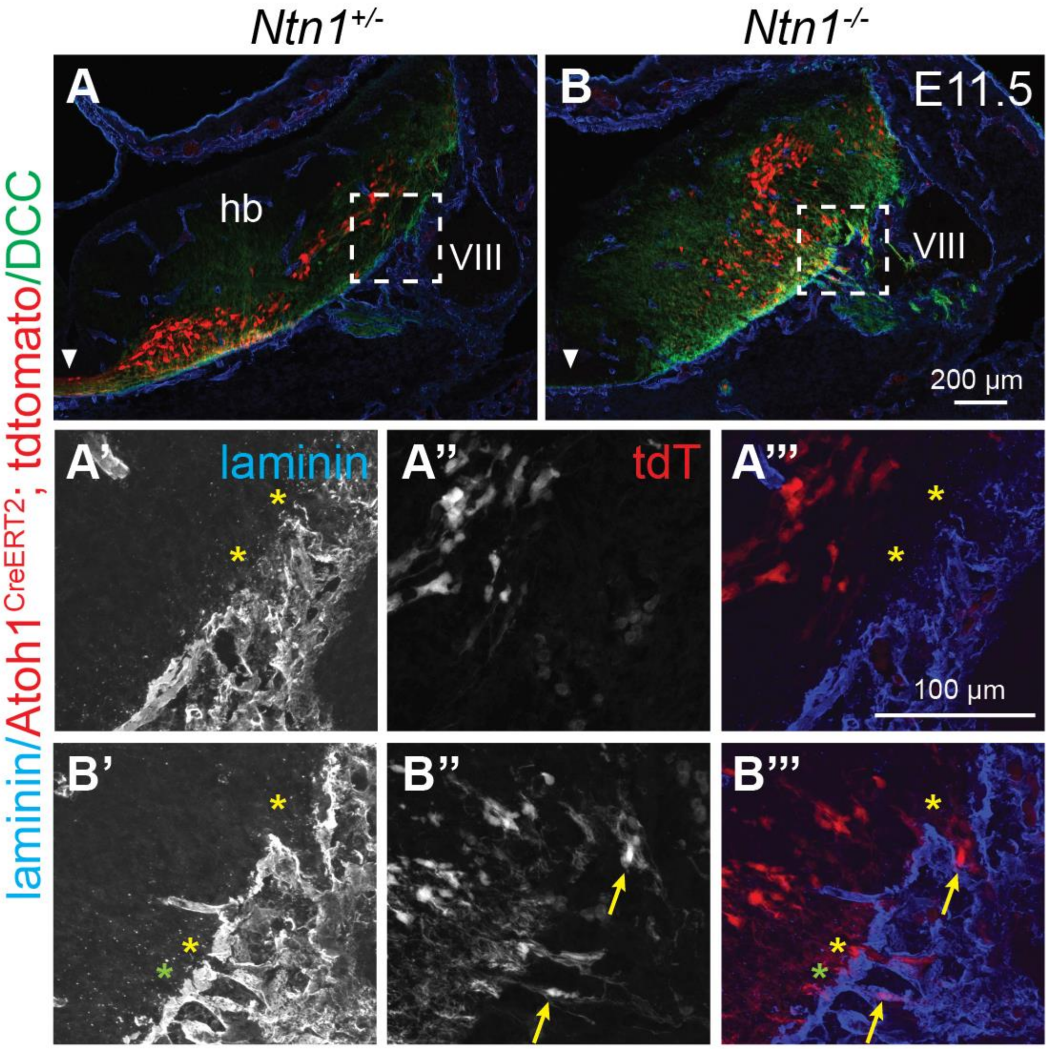

Figure S1: Earlier born rhombic lip neurons exit the CNS at cranial nerve roots in the absence of *Ntn1*. Related to Figure 2. (A-B''') Immunostains of E11.5 transverse head sections from animals that were injected with tamoxifen at E9.5. Low power images of laminin (blue) and tdTomato (red) show that in the absence of Ntn1, commissural neurons fail to form a ventral commissure (white arrowhead) and are located more dorsally (A, B). In control animals (A-A'''), tdTomato+ neurons do not take advantage of weaker areas of BM integrity near nerve entry zones (yellow asterisks, A'-A'''). In contrast, in mutants (B-B'''), a number of processes and cell bodies are observed migrating through the BM (yellow arrows), generating *de novo* breaks in laminin (yellow asterisks). In some cases, neurons traverse the BM before a clear break is observed (green asterisk, B'-B'''). Hb, hindbrain; VIII, vestibulocochlear nerve.

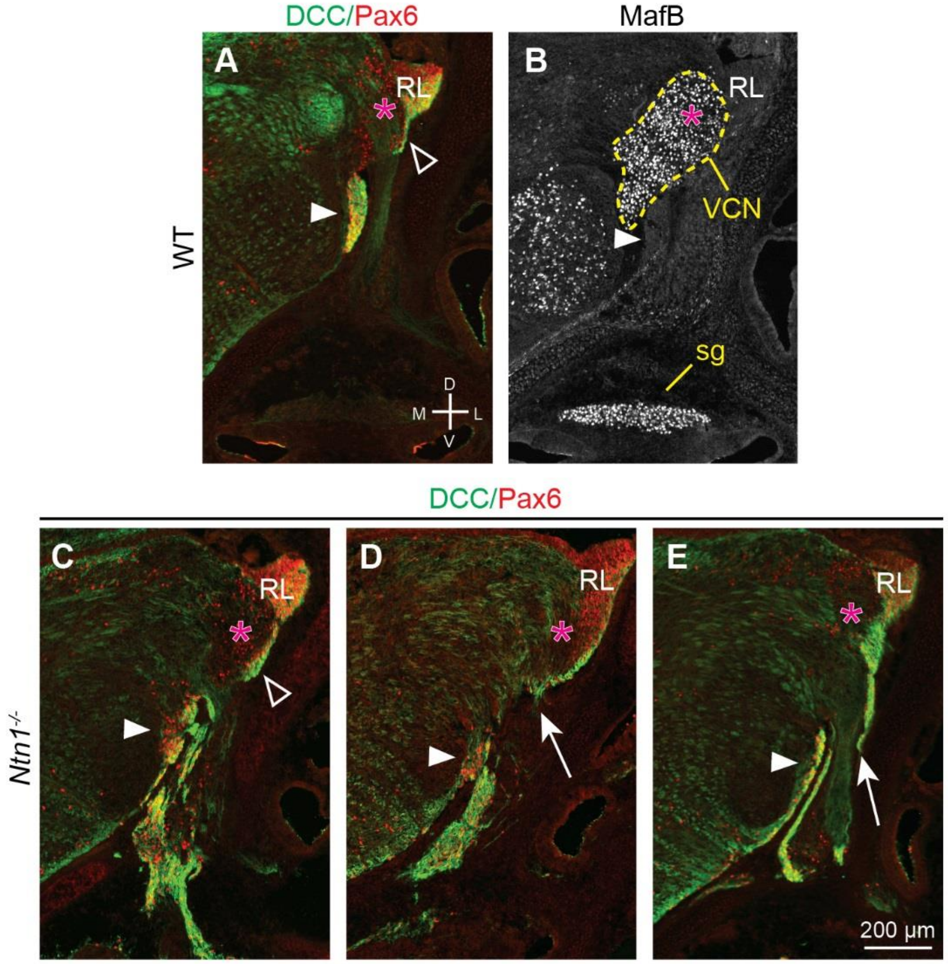

Figure S2: PNs exit the CNS independently of other populations of neurons. Related to Figure 2. (A-E) E15.5 transverse sections of the embryonic head immunostained for DCC (green, A, C-E) and Pax6 (red, A, C-E) or for MafB (B), a marker of earlier born ventral cochlear nucleus (VCN) neurons (Howell *et al.,* 2007). Comparisons between anatomically similar WT sections (A, B) reveal that the MafB+ VCN (*; outlined in B) lies ventromedial to the DCC/Pax6+ secondary rhombic lip (RL) and dorsal to the DCC/Pax6+ AES (filled arrowhead). In 5 out of the 9 *Ntn1*$^{-/-}$ embryos examined, DCC+ VCN axons appear confined to the CNS (C, hollow arrowhead) as in WT animals (A, hollow arrowhead). In the remaining 4/9 animals, VCN axons were seen projecting along the outside of the VIIIth nerve to varying degrees (D, E; arrows). However, in all cases, AES neurons exited the CNS and followed a path that was distinct from the occassional ectopic VCN axons, which departed via the more medial aspect of the VIIIth nerve. AES, anterior extramural stream; RL, secondary rhombic lip; sg, spiral ganglion; *, VCN.

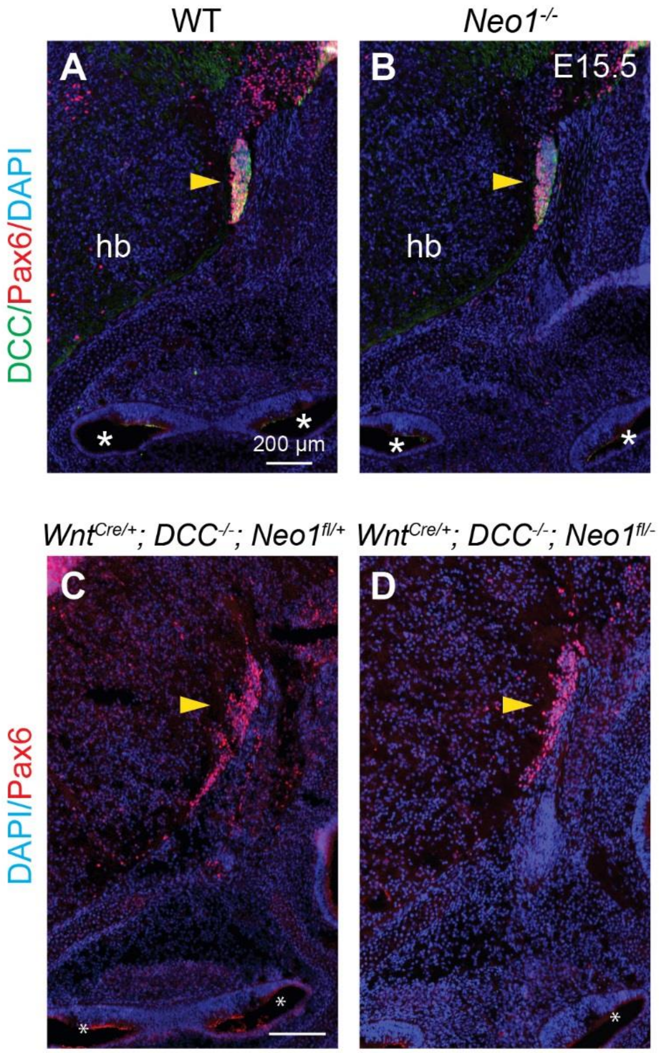

Fig. S3: PNs migrate normally in complete *Neo1* null mutants, and loss of *Neo1* selectively from the rhombic lip does not enhance CNS departure in *DCC*$^{-/-}$ animals. Related to Figure 4. (A-D) Immunostains in E15.5 receptor mutants. The AES (yellow arrowhead), indicated by Pax6 (red) and DCC (green, A-B) immunoreactivity, looks grossly normal in complete *Neo1* nulls (A, B). Loss of *Neo1* specifically in rhombic lip precursors (D) did not greatly enhance the *DCC* single mutant phenotype (C). In both cases the AES is semi-intact, and there are a smattering of neurons residing outside of the CNS. Many of these Pax6+ neurons were still located within the vestibulocochlear nerve and had not yet traveled to the cochlea proper. Hb, hindbrain; *, cochlear duct.

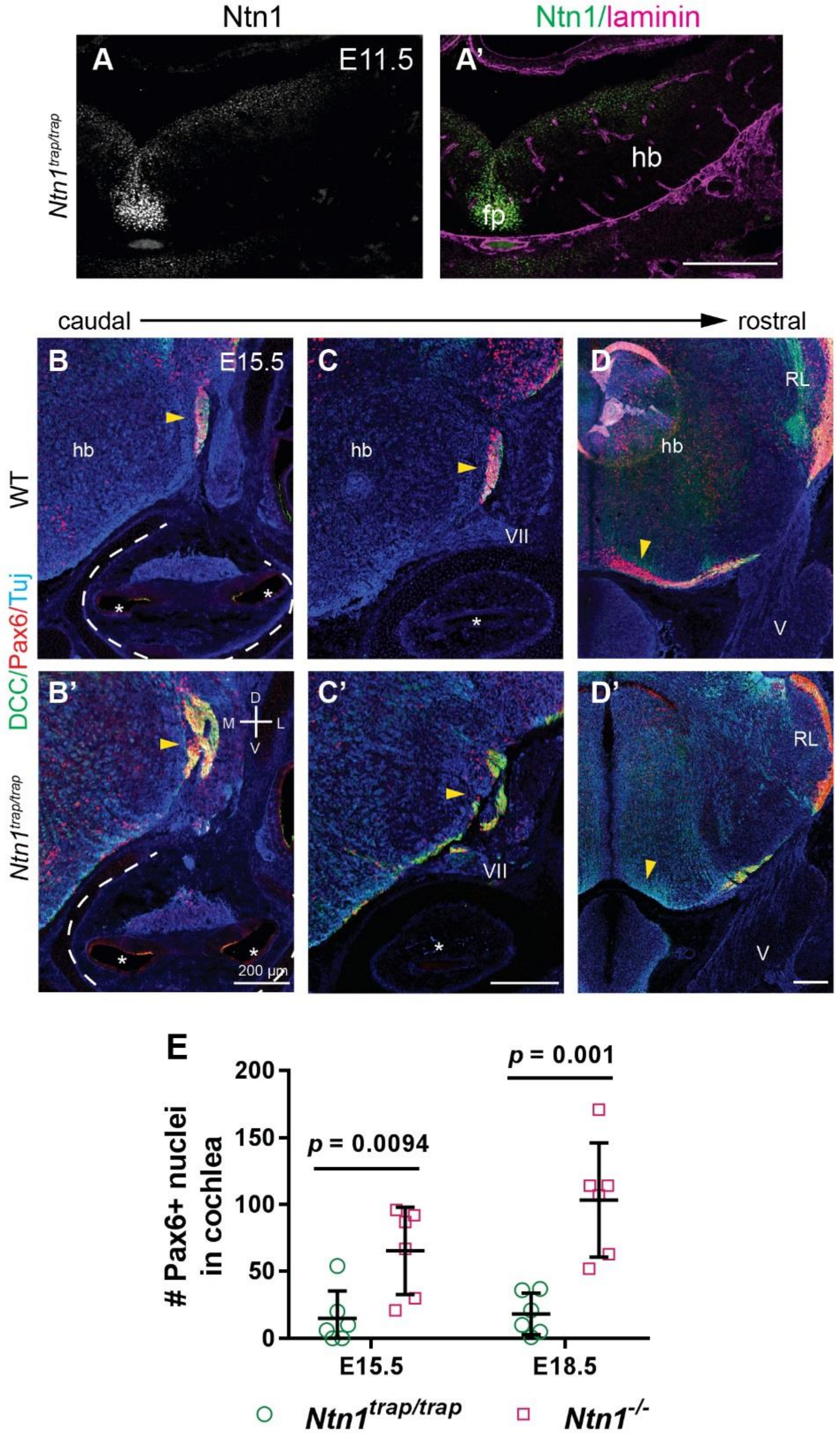

Ntn1
Ntn1/laminin
A
E11.5
A'
Ntn1trap/trap
hb
fp
caudal
rostral
B
E15.5
C
D
hb
hb
hb
RL
WT
VII
V
DCC/Pax6/Tuj
B'
C'
D'
D
M
L
V
RL
Ntn1trap/trap
VII
V
200 µm
E
p = 0.001
p = 0.0094
# Pax6+ nuclei in cochlea
200
150
100
50
0
E15.5
E18.5
Ntn1trap/trap
Ntn1-/-

Fig. S4: Fewer PNs exit the CNS in hypomorphic *Ntn1* mutants. Related to Figure 6. (A-D') Immunostains of transverse sections of *Ntn1*$^{trap/trap}$ embryonic heads. (A-A') Ntn1 (green) and laminin (magenta) immunostaining at E11.5 shows an absence of Ntn1 at the SPR and puncta reflecting trapped Ntn1 fusion protein at the floor plate and ventricular zone. (B-D') Immunostaining for Tuj (blue), Pax6 (red) and DCC (green) at E15.5 reveal ectopic PNs in hypomorphs (B'-D'). Compared to the intact AES along the sub-pial region in WT animals (B-D, yellow arrowheads), the stream of migrating PNs appears disrupted in *Ntn1*$^{trap/trap}$ animals, with some departing into the periphery at the level of the VIIIth (B') and VIIth (C') nerve roots. Many PNs get close to the midline (D'), which is rarely observed in complete nulls (see Fig. 2), but the pontine nuclei still fail to form. (E) Quantification of ectopic Pax6+ neurons in the base and middle turns of the cochlea in hypomorphic vs. complete null mice (mean ± S.D., Student's t-test). Fp, floor plate; hb, hindbrain; V, trigeminal nerve; VII, facial nerve.

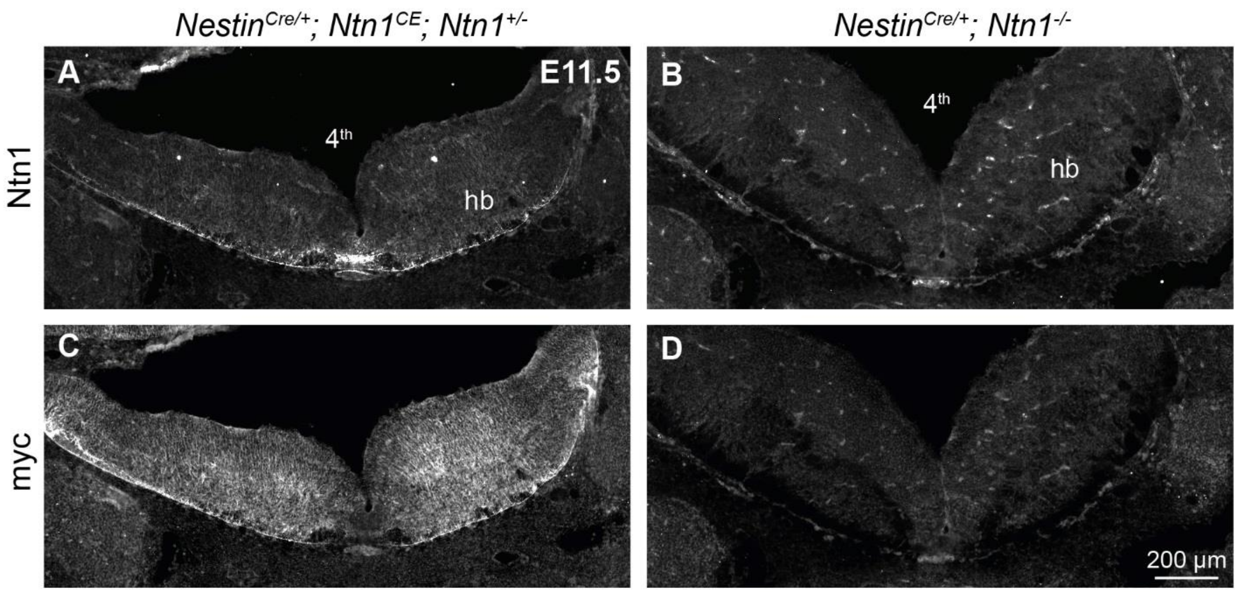

Fig. S5: Ntn1 and myc antibodies are epitope-specific. Related to Figure 7. (A-D) Immunostains of transverse sections of E11.5 embryonic heads. (A-B) Ntn1 immunostaining is eliminated in *Ntn1*$^{-/-}$ animals. (C-D) Myc immunostaining is only present in the presence of Cre and the *Ntn1* conditional expressor allele. 4th, fourth ventricle; hb, hindbrain.

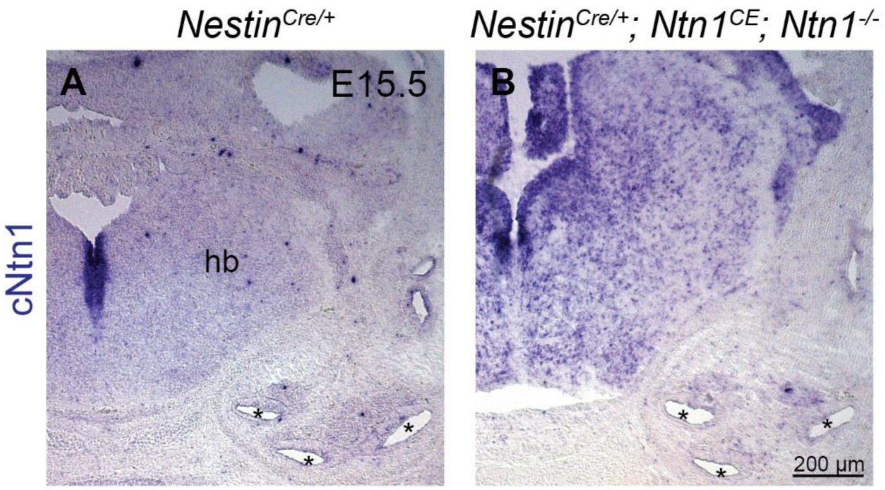

Fig. S6: *Nestin*^{Cre} drives broad *cNtn1* expression in the hindbrain. Related to Figure 7. (A-B) *In situ* hybridization for *cNtn1* shows cross-reactivity with endogenous *mNtn1* at the midline (A); *cNtn1* expression expands throughout the rest of the hindbrain in the presence of the conditional allele (B). Hb, hindbrain; *, cochlear ducts.

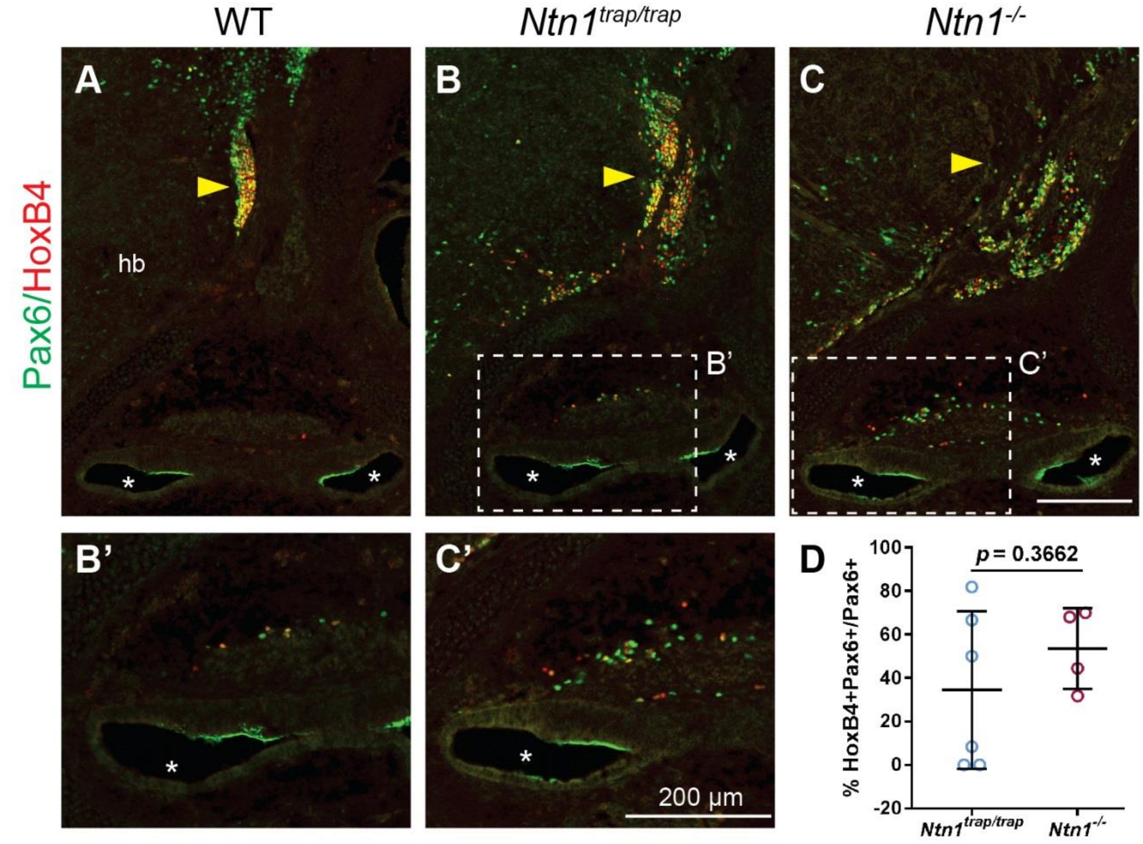

Fig. S7: PN subsets do not preferentially exit the CNS in *Ntn1* mutants. Related to Figure 4. (A-C) E15.5 transverse embryonic head sections immunostained for Pax6 (green), which labels all PNs, and HoxB4 (red), which labels subsets of PNs in a dorsoventral gradient (dorsal low, ventral high) (di Meglio et al., 2013). When compared to WT animals (A), *Ntn1* hypomorphs appear to retain this gradient of expression in remnants of the AES (B), indicating that the identity of PN subsets are preserved. We find that both HoxB4+ and HoxB4- subsets of PNs exit the CNS when Ntn1 levels are reduced (B) or eliminated (C), and they appear in the base of the cochlea at similar rates (B', C'), quantified in (D) (mean ± S.D., Student's t-test). Hb, hindbrain; *, cochlear duct.

Supplemental Experimental Information

Immunohistochemistry

Embryos were fixed in 4% paraformaldehyde (PFA)/PBS at 4°C overnight, cryoprotected in sucrose, frozen in NEG-50 (Thermo-Scientific), and sectioned at 12 to 16 µm. Sections were blocked in 3% BSA and incubated in the following primary antibodies at 4°C overnight: 1:500 goat anti-DCC (Santa Cruz), 1:750 rabbit anti-laminin (Sigma), 1:500 rabbit anti-MafB, 1:250 rat anti-myc (Santa Cruz), 1:500 goat anti-Netrin-1 (R&D), 1:400 goat anti-Neogenin (R&D), 1:400 rabbit anti-Pax6 , 1:100 mouse anti-RC2 (DSHB), 1:100 goat anti-TAG1 (R&D), and 1:1000 mouse anti-Tuj1 (Covance). For antigen retrieval, the sections were treated with boiling 10 mM sodium citrate, pH 7.0, for 20 minutes prior to blocking. Species-specific secondary antibodies conjugated to Alexa-Fluor fluorophores from Jackson ImmunoResearch or Invitrogen were used afterward.

Whole-mount brains were dissected and fixed in 4% PFA/PBS at 4°C overnight and blocked in 10% normal donkey serum (NDS) and 1% Triton-X in PBS at 4°C overnight. After incubating in primary antibody for 3 nights, the brains were incubated in HRP-conjugated secondary antibodies. Detection was performed using a DAB substrate.

In situ *hybridization*

Standard *in situ* hybridization was performed as described on 12 µm sections (Abraira et al., 2008). The *Egr2* probe was provided by Advanced Cell Diagnostics (ACD, Hayward, CA) for use with their RNAscope Fluorescent Multiplex Kit. Tissue sections were rinsed with PBS to wash off residual Neg-50 and treated with protease III (ACD) before following the manufacturer's protocol.

Transmission electron microscopy (TEM)

Embryos were collected and rinsed in 0.1 M sodium cacodylate buffer before drop-fixing in a modified Karnovsky fixative (2.5% paraformaldehyde, 5% glutaraldehyde, and 0.06% picric acid in 0.2 M cacodylate buffer) (Ito and Karnovsky, 1968) for 3-5 days at 4˚C. Whole fixed embryos were then embedded in epon resin, and ultrathin sections of 80 nm were collected on copper grids and counter-stained with Reynold's lead citrate (0.2% lead citrate).

Imaging

Images were collected on an Olympus VS120 slide scanner at 10X and 20X. Higher power images were taken with an Olympus Fluoview 1200 at 40X or a Leica SP8 confocal microscope at 25X or 40X. A 1200EX electron microscope (JEOL) equipped with a 2k CCD digital camera (AMT) captured all TEM images. Images were processed using ImageJ (NIH) and Adobe Photoshop.

Image Quantification

To quantify the number of Pax6+ nuclei in the cochlea, we counted the number of labeled cells in the base and middle turns of the cochlea in the earliest section where all three turns of the cochlea are first visible. To perform the same analyses in the VIIth nerve root, we only used sections where the nerve could be seen exiting the hindbrain to be confident of the anatomy. We counted all cells present in a 110 x 218 pixel box over this initial segment of the VIIth nerve.

The area covered by laminin was measured by centering a 561 x 386 pixel box over the BM adjacent to the AES, with the top of the box meeting the dorsal tip of the BM where it stops to permit the entry of the VIIIth nerve. After thresholding, the area covered by laminin within the box was calculated.

Ntn1 intensity was measured using confocal images at the floor plate or at the lateral edges of the hindbrain. The z-stack was summed and converted to an 8-bit image, and a 158 μm^2 circle was placed at the floor plate or at the basement membrane immediately ventral to the VIIIth nerve root. If a blood vessel was present, the circle was placed at the next available area. Each data point in the figure represents the mean intensity found over the area of the circle. All image analyses were performed using ImageJ (NIH).

Supplemental References

Ito, S., and Karnovsky, M. (1968). Formaldehyde-glutaraldehyde fixative containing trinitro compounds. J. Cell Bio. *39*, 168–169.
